# Periostin/Filamin-A: A Candidate Central Regulatory Axis for Valve Fibrogenesis and Matrix Compaction

**DOI:** 10.3389/fcell.2021.649862

**Published:** 2021-06-03

**Authors:** Suniti Misra, Shibnath Ghatak, Ricardo A. Moreno-Rodriguez, Russell A. Norris, Vincent C. Hascall, Roger R. Markwald

**Affiliations:** ^1^Department of Biochemistry and Molecular Biology, Hollings Cancer Center, Medical University of South Carolina, Charleston, SC, United States; ^2^Department of Regenerative Medicine and Cell Biology, Medical University of South Carolina, Charleston, SC, United States; ^3^Department of Biomedical Engineering/ND20, Cleveland Clinic, Cleveland, OH, United States

**Keywords:** periostin, α5β1-integrin, valve interstitial cushion cells, Fak, Pak1, Cdc42, actin remodeling, filamin A

## Abstract

**Background:**

Discoveries in the identification of transcription factors, growth factors and extracellular signaling molecules have led to the detection of downstream targets that modulate valvular tissue organization that occurs during development, aging, or disease. Among these, matricellular protein, periostin, and cytoskeletal protein filamin A are highly expressed in developing heart valves. The phenotype of periostin null indicates that periostin promotes migration, survival, and differentiation of valve interstitial cushion cells into fibroblastic lineages necessary for postnatal valve remodeling/maturation. Genetically inhibiting filamin A expression in valve interstitial cushion cells mirrored the phenotype of periostin nulls, suggesting a molecular interaction between these two proteins resulted in poorly remodeled valve leaflets that might be prone to myxomatous over time. We examined whether filamin A has a cross-talk with periostin/signaling that promotes remodeling of postnatal heart valves into mature leaflets.

**Results:**

We have previously shown that periostin/integrin-β1 regulates Pak1 activation; here, we revealed that the strong interaction between Pak1 and filamin A proteins was only observed after stimulation of VICs with periostin; suggesting that periostin/integrin-β-mediated interaction between FLNA and Pak1 may have a functional role *in vivo*. We found that FLNA phosphorylation (S2152) is activated by Pak1, and this interaction was observed after stimulation with periostin/integrin-β1/Cdc42/Rac1 signaling; consequently, FLNA binding to Pak1 stimulates its kinase activity. Patients with floppy and/or prolapsed mitral valves, when genetically screened, were found to have point mutations in the filamin A gene at P637Q and G288R. Expression of either of these filamin A mutants failed to increase the magnitude of filamin A (S2152) expression, Pak1-kinase activity, actin polymerization, and differentiation of VICs into mature mitral valve leaflets in response to periostin signaling.

**Conclusion:**

PN-stimulated bidirectional interaction between activated FLNA and Pak1 is essential for actin cytoskeletal reorganization and the differentiation of immature VICs into mature valve leaflets.

## Introduction

Congenital heart disease (CHD) is an important cause of child morbidity and mortality worldwide, and genetic mutations have important roles not only in the developmental anomalies but also as an underlying “hidden” contributor to adult myocardial hypertrophic remodeling diseases and myxomatous degeneration associated with prolapse or calcific valve stenosis. The prevalvular mesenchyme of both the inlet [atrioventricular (AV)] and outlet (arterial) valves originates by the transformation of endocardium into cushion mesenchyme; however, the mesenchyme does not fully differentiate into mature leaflets or cusps until after birth. Their subsequent elongation and compaction into mature leaflets are a normal postnatal event characterized by the progressive alignment of immature valvular progenitor (cushion) cells into differentiated, mature valve interstitial cells (VICs). The failure to complete remodeling or “compaction” of the collagenous-rich matrix secreted by VICs results in enlarged, truncated leaflets in which the extracellular matrix remains unorganized, and the progenitor cells remain poorly differentiated or are abnormally differentiated (e.g., into osteogenic, chondrogenic, or cardiomyocyte lineages) (Butcher and Markwald, 2007; Butcher et al., 2007a; Norris et al., 2008a, b; Snider et al., 2008), and they are mechanically weakened (Butcher et al., 2007a). The resulting “floppy” valves are prone to prolapse and unable to consistently sustain normal hemodynamics throughout adult life (Markwald et al., 2010). This can result over time in a reversal (regurgitation) of flow, particularly when challenged by environmental stressors or following cardiac injuries such as an infarct (Levine et al., 2015). For example, 1 in 40 of the United States population will experience in their lifetime a mitral valve prolapse (Durst et al., 2015). Even more frequently, abnormalities in embryonic or postnatal valvulogenesis and remodeling can present later in life as myxomatous leaflets or calcific stenosis of the aortic valve, indicating that there is a developmental link or origin for some adult cardiovascular diseases (Lauriol et al., 2016). Thus, the clinical relevance of abnormal valvulogenesis is not limited to visible structural defects at birth, like bicuspid aortic valves, hypoplastic or missing leaflets, or parachute mitral valve leaflets, but also to hidden defects that predispose the leaflets to degenerative processes (Enriquez-Sarano et al., 2005; Nkomo et al., 2006) that can exacerbate cardiac injuries and lead to prolapse and regurgitation as well as contributing indirectly to arrhythmias or cardiac failure.

We have previously shown that the matricellular protein periostin (PN) is a key regulator of valve morphogenesis that functions postnatally to complete remodeling of hypertrophied, thickened primordia into attenuated and compact, mature leaflets (Norris et al., 2009a, b; Tkatchenko et al., 2009). Evolutionarily, PN is homologous to ancient adhesion proteins called fasciclins that have important roles in tissue formation and patterning (Lindsley et al., 2005; Norris et al., 2008a, b). However, it is unclear how PN actually functions in postnatal valvulogenesis and maturation. As a matricellular protein, there are at least two ways that PN could coordinate valvulogenic tissue remodeling via 3D collagen matrix compaction. It can directly bind and cross-link extracellular matrix fibrils, especially collagen, assuming that this protein is being properly synthesized and secreted by valve cells (Snider et al., 2008). Also, it can bind directly to cell surface integrin receptors (Ghatak et al., 2014), which can activate intracellular signaling kinases, e.g., Akt, PI3K or Erk, and by an α5β1 integrin/focal-adhesion-kinase (Fak/Akt1) pathway in valve progenitor cells (Ghatak et al., 2014). Periostin binding to α5β1 integrin promoted collagen secretion and the activation of hyaluronan synthase 2 (Has2) to increase synthesis and secretion of hyaluronan and activation of CD44, a hyaluronan receptor on many cell types, including fetal VICs (Ghatak et al., 2014), and it did so in a time frame that correlated with the elongation of developing prenatal AV cushions. Interestingly, a positive feedback loop between PN, PI3K, and hyaluronan/CD44 sustains PN expression, and increases phosphoserine-HAS2 expression and hyaluronan production (Ghatak et al., 2014). In addition to promoting hyaluronan synthesis, PN also promotes cell migration in 3D culture assays. Transfection of embryonic day 15.5 mice AV cushion valve progenitor cells with full-length PN cDNA enhanced fourfold their migratory behavior, whereas migration was blocked or inhibited by transfection with PN or FAK-silencing vectors or by adding either inhibitors of PI3K/AKT kinase activity or β-integrin-blocking antibodies to the medium. Silencing Erk/Map kinase did not affect cushion cell migration but did reduce their expression of collagen (Ghatak et al., 2014, 2019).

Filamin A (FLNA) is a 2,647-amino acid-long actin-crosslinking protein, with 2 N-terminal actin-binding domains followed by 24 immunoglobulin-like repeats (Ithychanda et al., 2009). FLNA is mostly located in the membrane cytoskeleton, underneath the plasma membrane, where it regulates a variety of cytoskeleton-related processes, including receptor clustering and cross talk among different receptors and the actin cytoskeleton (Ithychanda et al., 2009). By binding to the actin cytoskeleton, FLNA changes its organization and association with cell surface membrane proteins that interact with extracellular structural proteins including collagen (Weihing, 1988; Washington and Knecht, 2008; Duval et al., 2014, 2015; Mohammadi et al., 2015). FLNA can be phosphorylated by protein kinase A (PKA) (Wallach et al., 1978a, b), including FLNA phosphorylation at Ser2152, located in the Ig20 repeat. Phosphorylation of FLNA promotes many cellular functions, including cytoskeleton remodeling and cell (e.g., platelets) migration (Ithychanda et al., 2015). FLNA S2152 phosphorylation is specifically required for Pak1-mediated actin cytoskeletal assembly (Vadlamudi et al., 2002). Mutations in the FLNA gene have recently been identified in multiple families with an X-linked form of myxomatous valvular dystrophy (Kyndt et al., 1998, 2007; Lardeux et al., 2011; Duval et al., 2014, 2015). Our recent study (Ghatak et al., 2019) showed that PN induced β1 integrin/FAK(Y397)/PI-3 kinase (PI3K)/Akt1 that links with mTOR/p70S6K to regulate Pak1-mediated cell survival and actin reorganization in VICs. Moreover, Akt signaled both downstream and upstream of Pak1 in VICs and, in both scenarios, promoted VIC survival by protecting them from apoptosis (Ghatak et al., 2019). However, the mechanism(s) by which FLNA is regulated by upstream signaling intermediates such as PN to promote fibrogenic differentiation and remodeling of pleural- postnatal valves into mature leaflets is unclear and are the focus of this study.

Here, we explored the potential of using the PN/Integrin β1 signaling through Cdc42/Pak1 interaction as a way not only to stimulate polymerized actin cytoskeleton formation with higher F-actin/G-actin ratios but also to stimulate association of Pak1 with FLNA for FLNA activation and support the integrity of actin cytoskeletal assembly. Our study shows that PN/Integrin β1 can activate Pak1 kinase through two signaling mechanisms: (1) Our previous work demonstrated that a PN/integrin β1/PI3K/S6K pathway activating Pak1 kinase regulates actin polymerization in VICs (Ghatak et al., 2019). (2) This present study demonstrates that Cdc42 and Rac accumulate in activated GTP-bound forms in response to PN/Integrin β1/Fak/Src interaction and that Cdc42 associates with the actin cytoskeleton through activation of Pak1 kinase, whereas Rac1 relocalizes to the migratory front in VICs. The importance of our previous (Ghatak et al., 2019) and present studies are that these two different signals lead to activation of Pak1 (Thr 423), and these pathways promote binding of Pak1 to full-length FLNA and its subsequent phosphorylation at S2152 and resulting in the binding of FLNA to Pak1 to stimulate its kinase activity. Collectively, these results indicate that PN-regulated, bidirectional Pak1–FLNA interactions may influence the actin cytoskeletal rearrangements and promote remodeling of postnatal heart valves into mature leaflets. Patients with floppy and/or prolapsed mitral valves, when genetically screened (as part of an international Leducq network), were found to have point mutations in the FLNA gene at P637Q, and G288R (Kyndt et al., 2007; Dina et al., 2015; Duval et al., 2014, 2015). Either of these mutations resulted in the inhibition of Rac1 activation and the phosphorylation of FLNA at S2152, which correlated with changes in migration, actin cytoskeletal assembly, and differentiation of VICs. These findings provide evidence for the potential clinical relevance of the phosphorylation of FLNA at S2152 in valve remodeling by PN downstream signaling targets Cdc-42 and Pak1.

## Materials and Methods

### Samples and Study Design

Wild-type (WT) mice (C57BL/6 strain) were obtained from the Jackson Laboratory. PN-deficient mice on a C57BL/6 genetic background were provided by Dr. Simon Conway (Indiana University, Indianapolis, IN, United States). Mice at 8–10 weeks of age were used in all biochemical experiments as described previously (10). All animal care and experiments were done in accordance with the institutional guidelines. After removing the mitral valves from mice, the valves were minced and digested with 2 μg/ml of collagenase for 30 min at 37°C. Valve interstitial cushion cells were isolated as described previously (Castaldo et al., 2013). Briefly, cardiac valve samples were minced and then enzymatically disaggregated by incubation in 0.25% trypsin and 0.1% *(w/v)* collagenase II (both from Sigma-Aldrich) for 30 min at 37°C. The digestion was stopped by adding Hanks’ balanced salt solution supplemented with 10% fetal bovine serum (FBS). The tissue was further disaggregated by pipetting, and noncardiomyocyte cells were separated from debris and cardiomyocytes by sequential centrifugation and passage through a 20-μm-cell sieve. Fibroblastic VICs were isolated from cell suspension by immunomagnetic cell sorting through positive selection with Anti-fibroblast Microbeads (Miltenyi Biotech, Bergisch Gladbach, Germany). Following selection, VICs were cultured in Medium 199 (M199, Invitrogen) containing 5% fetal bovine serum (FBS), 0.5 ng/ml of EGF, 5 μg/ml of insulin, 2 ng/ml of bFGF, 100 U/ml of penicillin, and 100 μg/ml of streptomycin and incubated at 37°C with 5% CO_2_, 95% air. Materials: FBS was from Atlanta Biological, and L-glutamine, gentamicin sulfate, and amphotericin B were from Hyclone. Nonidet P-40, EGTA, sodium orthovanadate, glycerol, phenylmethylsulfonyl fluoride, leupeptin, pepstatin A, aprotinin, and HEPES) were purchased from Sigma.

### Agonists and Antibodies

Recombinant Human PN protein is from Abcam. Antibodies used in the entire study were either from Abcam, R&D Systems, Epitomic, Cell Signaling, or Santacruz Biotechnology. Anti-α5β1 integrin [Millipore Sigma, Cat. No. JBS5 and Abcam, Cat. No. (EP1041Y) (ab52971)] and anti-αVβ3 integrin (Millipore Sigma, Cat. No. LM609) are from Millipore Sigma and Abcam, respectively. Anti-integrin beta 5 [Abcam, Cat. No. (ab184312)] and α5 integrin antibody [Abcam, Cat. No. (ab78614)] are from Abcam. Anti-αVβ3 integrin (Chemicon, Cat. No. LM609) is from Chemicon. Anti-α5 [Biorbyt Cat. No. (orb469770)] and β3 [Biorbyt Cat. No. (orb457494)] antibodies are from Biorbyt. Ant-Rac1 (Thermo Fisher, Cat. No. PA1-091), anti-Cdc42 (Thermo Fisher, Cat. No. 10155-1-AP), anti-periostin (Abcam Cat. No. ab14041), anti-Filamin (Cell Signaling, Cat. No. 4762), anti p-Filamin (Ser2152) (Cell Signaling, Cat. No. 4761), and anti-Myc tag (Millipore Sigma, Cat. No. SAB4301136) antibodies are from Abcam, Thermo fisher, Cell Signaling, and Millipore Sigma.

### Immunohistochemical Staining in the Heart Sections

Heart sections from WT and PN-null mice were deparaffinized using standard procedures and permeabilized with 0.1% Triton X-100 in PBS. PN, filamin A, and phosphorylated filamin A (Ser2152) were localized in sections by immunohistochemical staining using appropriate antibodies following standard protocols. As a negative control, the primary antibody was replaced with nonimmune rabbit IgG (in such cases, no staining was observed).

### Plasmids

Pak1 T423E (Addgene plasmid # 12208), Pak1-H83L-H66L (pCMV6M-Pak1 H83L H86L) (Addgene plasmid # 12211), and Pak1-H83L-H66L-K299R (pCMV6M-PAK1 H83L H86L K299R) (Addgene plasmid # 26592) (Sells et al., 1997) were gifts from Jonathan Chernoff. Myc-FLNA S2152A (Add gene plasmid # 8983) and Myc-FLNA WT (Addgene plasmid # 8982) (Woo et al., 2004) were gifts from John Blenis. DN-N17Rac1 (Add gene plasmid # 20151) (Inoue et al., 2005) was a gift from Tobias Meyer. pGEX-DN-Pak1 (aa 83-149) (Addgene plasmid # 12216) (Xiao et al., 2002) was a gift from Jonathan Chernoff.

cDNAs encoding Pak1 were subcloned into the epitope-tagged expression vector pJ3M (Sells and Chernoff, 1995) and then subcloned into a modified version of pCMV5 (Andersson et al., 1989). For some experiments, an amino-terminal Myc epitope-tagged Pak1 was used. Similarly, constructs encoding FLNA were subcloned into the epitope-tagged (with GST and Myc) expression vectors pJ3M (Sells and Chernoff, 1995), and then subcloned into pGEX5X (Amersham) and pcDNA3.1 (Invitrogen) vectors. The FLNA G288R or FLNA P637Q (Kyndt et al., 1998), or DN D57Y Cdc42 (Stowers et al., 1995) mutations were generated by site-directed mutagenesis using Myc-FLNA and Cdc42 as backbone constructs and the Quick Change mutagenesis kit (Stratagene), followed by sequencing to confirm mutations. VICs were serum starved for 24 h and then transfected with shRNAs for control [non targeted shRNA (NT shRNA)] and shRNA for the targeted protein for 12 h, and then the transfectants were incubated in complete medium for another 36 h.

### shRNAs

The full-length coding nucleotide sequences of the periostin, Pak1, S6K, and Filamin derived from the NIH website^1^ were used to design two shRNA sequences using the program at the portals.broadinstitute.org.

#### Periostin shRNA #1

Sense oligo:5′-TGCTTATTGTTAACCCTATAAATTCAAGAGATTTATAGGGTTAACAATAAGCTTTTTTC-3′

Antisense oligo:5′-TCGAGAAAAAAGCTTATTGTTAACCCTATAAATCTCTTGAATTTATAGGGTTAACAATAAGCA-3′

#### Periostin shRNA #2

Sense oligo:5′-TATCCATGGAGAGCCAATTATTTTCAAGAGAAATAATTGGCTCTCCATGGATTTTTTTC-3′

Antisense oligo:5′-TCGAGAAAAAATCCATGGAGAGCCAATTATTTCTCTTGAAAATAATTGGCTCTCCATGGATA-3′

#### S6K shRNA #1

Sense oligo:5′-TGTA GCATGGAACATTG TGAGAAATTTCAAGAGAATTTCTCACATTTCTCACAATGTTCCATGCTTTTTTC-3′

Antisense oligo:5′-TCGAGAAAAAAGCATGGAACATTCGTGAGAAATTCTCTTGAAATTTCTCACAATGTTCCATGCTACA-3′

#### S6K shRNA #2

Sense oligo:5′-TGTATTTGCCATGAAGGT GCTTAAA TTCAAGAGATTTAAGCACCTT CATGGCAAA TTTTTTC-3′

Antisense oligo:5′-TCGAGAAAAAATTTGCCATGAAGGAGCTTAAATCTCTTGAATTTAAGCACCTTCATGGCAAATACT-3′

#### Pak1 shRNA #1

Sense oligo:5′-TGTAAGTCAGCTGAAGATTATATGTAAGTCAGCTGAAGATTATAATTTTCAAGAGAAATTATAATCTTCAGCTGACT TTTTT TC-3′

Antisense oligo:5′-TCGA GAAAAAAAGTCAGCTGAAGATTATAATTTCTCTTGAAAATTATAATCTTC AGCTGACTTACA-3′

#### Pak1 shRNA #2

Sense oligo:5′-TGTACCGAAGAAAGAGCTGATTATTTTCAAGAGAAATAATCAGCTCTTTCTTCGGTTTTTTC-3′

Antisense oligo:5′-TCGAGAAAAAACCGAAGAAAGAGCTGATTATTTCTCTTGAA AATAATCAG CTCTTTCTTCGGTACA-3′

#### FLNA shRNA #1

Sense oligo:5′-TGTACATGCGTATGTCCCACCTAAATTCAAGAGATTTAGGTGGGACATACGCATGTTTTTTC-3′

Antisense oligo:5′TCGAGAAAAAACATGCGTATGTCCCACCTAAATCTCTTGAATTTAGGTGGACATACGCATGTACA3′

#### FLNA shRNA #2

Sense oligo:5′-TGTAGGCCAACGTTGGTAGTCATTGTTCAAGAGACAATGACTACCAACGTTGGCCTTTTTTC-3′

Antisense oligo:5′-TCGAAAAAAAGGCCAACGTTGGTAGTCATTGTCTCTTGAACAATGACTACCAACGTTGGCCTACA-3′

The double stranded oligos were cloned at the HpaI–XhoI site in pSico (a gift from Tyler Jacks, Addgene plasmid #11578) and pSicoR (a gift from Tyler Jacks, Addgene plasmid #11579) vectors (Ventura et al., 2004): http://web.mit.edu/jacks-lab/protocols/pSico html.

### Lentiviral Transfections and Isolation of the Cell Lysates

All transductions of the indicated constructs were done by lentiviral constructs (at the indicated MOIs) in the serum-free (SF) medium 199 supplemented with 20% conditioned medium (CM) for 24 h, and then grown for another 48 h with medium 199 supplemented with 5% bovine serum albumin (BSA). Exogenous addition of 5 μg/ml PN in all experiments was done in the SF + 20% CM, and this addition of soluble protein was done to the culture medium of adherent VICs in the tissue culture plate. For transfections, isolated VICs (seeded at 1 × 10^6^ cells/100-mm dish) were grown to 70–80% confluence and then transfected with 10^5^ transformation unit (TU) lenti-virus-encoded plasmid DNAs using polybrene transfection protocol (Sigma Aldrich). The cells were allowed to express the protein for 48 h after transfection, then washed with phosphate-buffered saline (PBS). The lysis buffer [150 mM NaCl, 1% Nonidet P-40 (NP-40), 25 mM Tris-HCl, pH 7.5, 1 mM EDTA, 0.1 mM EGTA, 5 mM MgCl_2_, 1 mM dithiothreitol, 10% glycerol, 2% Nonidet P-40, 50 IU/ml aprotinin, 2 mg/ml leupeptin, and 1 mM phenylmethylsulfonyl fluoride] was added to each plate, and the transfected VICs were scraped into Eppendorf tubes at 4°C. After 5 min, the lysates were centrifuged at 14,000 rpm for 10 min, and the supernatants were collected.

### Quantitative Real-Time PCR

Total RNA was isolated from VICs after various treatments and transfections as mentioned in the figure legends for each specified experiment using the RNeasy Mini kit (Qiagen, United States) according to the standard protocol provided by the manufacturer, with on-column DNA digestion. RNA integrity and concentration were analyzed using Bioanalyzer (City State), and 1 μg of RNA was reverse transcribed into cDNA using the First Strand cDNA Synthesis kit from Roche (Qiagen, United States). SYBR-Green (Bio-Rad Biosystems) was used for all QPCR assays. Amplification was done with the QPCR analyzer (Bio-Rad Biosystems). The PCR reaction mixture (25 μl) contained 12.5 μl of 2x SYBR Green PCR Master Mix (Bio-Rad Biosystems), 5 μl of diluted RT product (1:20), and 0.5 μM sense and antisense primer sets. The QPCR assays were done in three individual experiments with triplicate samples using standard conditions. After sequential incubations at 50°C for 2 min and 95°C for 10 min, respectively, the amplification protocol consisted of 50 cycles of denaturing at 95°C for 15 s, annealing, and extension at 60°C for 60 s. The standard curve was made from a series dilution of template cDNA. Expression levels of PN were calculated after normalization with the housekeeping gene GAPDH.

### Primers for QPCR

#### PN

Forward primer:

5′-TGCTGCCCTGGCTATATGAG-3′ and

Reverse primer:

5′-GTAGTGGCTCCCACAATGCC-3′.

#### GAPDH

Forward primer:

5′-AGAACATCATCCCTGCATCC-3′ and

Reverse primer:

5′-CAGTGAGCTTCCCGTTCAGC-3′.

#### Pak1

Forward primer:

5′-CCACTTCCTGTTACTCCAACTC-3′ and

Reverse primer:

5′-GCTATCTGGCCTTCATCCATAC-3′.

#### S6K

Forward primer:

5′-TGACCCAGACCTTGCTTATTC-3′ and

Reverse primer:

5′-GGCAGTCAACGCTATAGTTAGT-3′.

### Production of GST-Fused Recombinant Proteins and Purification of Recombinant Pak1

The GTPase-binding domain (PBD) of human PAK1 (amino acids 67–150), PAK1 wild type (full length), and FLNA (amino acids 2,100–2,550) in fusion with GST were cloned into the bacterial expression vector pGEX-4T3. N17 Cdc42, D57Y Cdc42 (DN Cdc42), and empty vectors were inserted into pGEX-4T3. The GST–fusion proteins were purified using Glutathione Agarose 4B beads and stored at −80°C as a 50% suspension in 25 mM Tris-HCl [tris (hydroxymethyl) amino methane]-HCl, pH 7.4, 0.2 mM DTT (dithiothreitol), 1 mM MgCl_2_, and 5% glycerol]. Cells grown 24 h after seeding were lysed in lysis buffer. The cell lysates were centrifuged at 15,000 × *g* for 15 min at 4°C. Five hundred micrograms of cleared cell lysates was incubated with GST-tagged proteins (30 μg) and rotated (18 rpm) for 1 h at 4°C. The beads were washed four times with cell lysis buffer, and bound proteins were separated by SDS-PAGE. Bound Rac1, Cdc42, and RhoA were detected by immunoblotting as described in the specific experiments of figure legends.

### Affinity Precipitation Using GST–Fused Proteins

Precipitation of activated Rac1 or Cdc42 was done as described before (Benard et al., 1999). VIC lysates were prepared with the lysis buffer as described above in “Transfections.” Cytoskeletal and NP-40-soluble fractions were separated by centrifugation at 10,000 × *g* for 20 min. PBD-GST was used to trap Rac1-GTP or Cdc42-GTP in the 100-μl NP-40-soluble fraction. Incubation was done after addition of 200 μl of binding buffer (25 mM Tris-HCl, pH 7.4, 1 mM DTT, 30 mM MgCl_2_, 40 mM NaCl, 0.5% Nonidet P-40) for 1 h at 4°C. The bead pellets were then washed three times in binding buffer, twice with the same buffer without Nonidet P-40, and finally resuspended in 20 μl of Laemmeli sample buffer. Proteins were separated by 12% sodium dodecyl sulfate polyacrylamide gel electrophoresis (SDS-PAGE), transferred to nitrocellulose membrane, and blotted by using either monoclonal anti-Rac1 antibody or polyclonal anti-Cdc42 antibody. In some experiments, cytoskeletal and NP-40-soluble fractions were analyzed for the expression of Cdc42 by direct immunoblotting. As controls, lysates were incubated with 100 μM of either nonhydrolyzable GTPγS or guanosine diphosphate (GDP) before precipitation on PBD-GST beads, and both bead pellets and supernatants were analyzed. Immunoblots were analyzed with the enhanced chemoluminescence (ECL) kit from Amersham Pharmacia Biotech. GST-PAK1 WT was used to pull down PAK from the GST-N17 Cdc42, GST-N17 Rac1, and GST-D57Y Cdc42-transfected VICs that were lysed in solubilization buffer (50 mM HEPES, pH 7.4, 150 mM NaCl, 0.5% Triton X-100, 1 mM sodium fluoride, 10 mM β-glycerophosphate, 10 mM sodium pyrophosphate, 10 μg/ml of aprotinin, 5 μg/ml of leupeptin, 10 mM benzamidine, and 10 μg/ml of soybean trypsin inhibitor).

### Immunoprecipitation and Immunoblot

After transfection or treatments with the indicated constructs, or treatment with PN, VICs were lysed in solubilization buffer for 20 min on ice. Lysates were clarified by centrifugation at 4°C for 10 min at 10,000 × *g* rpm and precleared by incubation for 1 h at 4°C with 20 μl of protein G-Sepharose 4B packed beads. In some experiments, lysates were sonicated and incubated for 45 min at 37°C with DNase I (Sigma Aldrich). Immunoprecipitations were done for 2 h on ice with 5 μg/ml of the indicated antibodies. Immune complexes were collected on 25 μl of protein G-Sepharose 4B packed beads during 1 h at 4°C under agitation. Beads were washed three times in 1x solubilization buffer and twice in the same buffer containing 0.1% Triton X-100. Proteins were solubilized in Laemmli buffer, separated by SDS-PAGE, and transferred to nitrocellulose membranes for specific immunoblotting.

### PAK *in vitro* Kinase Assay

PAK was immunoprecipitated as described above, and GST-Pak1 was isolated from the VIC transfectants with indicated constructs, and from PN-treated lysates. Pak1 immunoprecipitates or GST-Pak1 were used for Pak1 enzyme activities. One microgram of purified myelin basic protein (MBP) or 1 μg of GST-FLNA was used as substrates. Dried beads were recovered in 1x kinase buffer (50 mM HEPES, pH 7.4, 10 mM MgCl_2_, 10 mM MnCl_2_, and 0.4 mM DTT). Purified myelin basic protein (MBP; 1 μg) was added, and the reaction was started by the addition of a mixture of 20 μM MgCl_2_-adenosine triphosphate (ATP) for 60 min for kinase enzyme assay. Nonradioactive assay kit (ab138879, Abcam) was used for kinase activity measurements. Data are presented as fold increase in Pak1 kinase activity with respect to vector control.

Kinase activity-tagged-Western blotting (KAT-WB) was done for autophosphorylation of serine/threonine residues. Total proteins of Pak1 enzyme (30 μg) were subjected to KAT-Western blotting as described earlier (Eto et al., 2020). Aliquots of an extract were loaded onto an SDS polyacrylamide gel, subjected to electrophoresis, and then proteins were transferred onto a sheet of PVDF membrane (0.2 μm). The membrane was sequentially treated with buffered 2-propanol, 6.0 M guanidine hydrochloride (Gu-HCl), 3.0 M Gu-HCl, 0.1 M Gu-HCl, and renaturation buffer. After an overnight renaturation step at 4°C, the membrane was soaked overnight at 4°C in the renaturation buffer supplemented with recombinant MBP as substrate or GST-FLNA as substrate at 0.2 mg/ml. Then the phosphorylation of substrates for kinase assay was determined after incubation with 1x kinase buffer for 60 min with 1 mM MgATP. The ratio of pMBP/total Pak1 or FLNA (S2152)/total Pak1 is presented as fold increase in Pak1 kinase activity with respect to control.

In some kinase assays, in addition to MBP, we also used 3 μg of purified FLNA in an *in vitro* kinase reaction using GST-Pak1 enzyme as above, 10 μCi of γ-^32P^ATP, and 25 μM cold ATP. Pak1 enzyme was removed by GST beads, and labeled FLNA was digested with calpain as described. The reaction was carried out in a volume of 30 μl for 30 min at 30°C and then stopped by adding 10 μl of 4× SDS sample buffer. The reaction products were analyzed by SDS-PAGE gel and autoradiography. Autophosphorylated PAK was visualized as a 65-kDa phosphoprotein, and its kinase activity was evaluated by phosphorylation of MBP (pMBP) or pFLNA (S2152).

In some experiments, the *in vitro* Pak1 kinase assays were further confirmed by nonradioactive assay kit (ab138879, Abcam), which is based on the monitoring of ADP formation. Pak1 kinase activity in this experiment is directly proportional to enzyme phosphotransferase activity and is measured fluorometrically by measuring fluorescence intensity with a fluorescence plate reader at excitation/emission = 540/590 nm. Data are presented as fold increase in Pak1 kinase activity with respect to control.

### Actin Polymerization Assays

The polymerization assay using pyrene actin (PA) was used as described previously (Doolittle et al., 2013; Henkels et al., 2015). VICs were either transfected with the relevant lentiviral expression plasmids for 48 h, or they were treated with PA for 10 min prior to harvesting. The PA was prepared in 0.5% fatty acid–free BSA in PBS. Cells were sonicated in actin lysis buffer (20 mM Tris-HCl, 20 mM NaCl, and 768 nM aprotinin). Ten microliters of cell lysates was added to 85 μl of pyrene-labeled actin-containing buffer, which was purchased as a kit (BK003) from Cytoskeleton, Incorporated (Denver, CO, United States). Actin polymerization buffer (10 μl) was added to the reaction for a final total volume of 105 μl. The enhanced fluorescence that occurs when pyrene G-actin (monomer) forms pyrene F-actin can be used to follow polymerization over time. Actin polymerization was measured for 12 min at 30-s intervals at the excitation of 350–360 nm with a bandwidth of 20 nm and at the emission of 401–411 nm with a bandwidth of 10 nm in a spectrophotometer microplate reader at room temperature. *In vitro* actin polymerization assay was done as outlined in the manufacturer’s (Cytoskeleton, Incorporated) instructions and following the procedure as described previously (Doolittle et al., 2013; Henkels et al., 2015).

### F/G-Actin Contents

Relative proportions of F-actin and G-actin were determined using a kit from Cytoskeleton (Denver, CO, United States). Blot images were scanned, and densitometry was measured using the software program NIH ImageJ.

### Confirming the Specificity of shRNA Experiments

To confirm the shRNA knockdown efficiencies in specific experiments, more than one shRNA was used. The knockdown experiments were confirmed by comparing the knockdown effects of shRNAs for coding sequences (CDS) with rescue of the observed shRNA-mediated knockdown phenotype by expression of a resistant form of the targeted mRNA. This was done: (1) by transfecting the cells with specific shRNAs for the CDS of the target gene; or (2) by cotransfecting the shRNA (CDS) for the target gene with or without corresponding cDNA transfection, or (3) by the indicated shRNA-mediated knockdown and the corresponding KI gene transfection as described (Ghatak et al., 2017a, b). Total cell lysates were examined by Western blot analysis for the indicated proteins. Total mRNAs were analyzed for the indicated mRNAs by RT-PCR and real-time PCR (QPCR). Because shRNAs were used to study PN-induced valve remodeling functions, several steps were followed as described in our recent papers (Ghatak et al., 2017a, b) and used in Figure 2E (inset), F to avoid the off-target problems and also confirm the specificity of the shRNAs used in this study. Specifically, the confirmation of knockdown by shRNAs was done by using more than one shRNA and demonstrating that the expression levels of the target protein and mRNA were substantially reduced, whereas the level of expression of an NT shRNA (scrambled shRNA)-transfected mRNA and/or protein was unaffected (Figure 2E (inset), F).

### Floating Collagen Gel Cultures and Quantitation of Gel Contraction

Experiments were done essentially as described previously (Ghatak et al., 2014). Briefly, 24-well tissue culture plates were precoated with BSA. For FLNA-induced effects, cells were pretransfected with vector control or WT FLNA expression vector, and FLNA mutants for transfectants were grown for 48 h. For Pak1 inhibition, the cells were treated with 2.5 μg/ml of IPA3 for 12 h. To determine the effect of PN, the transfected and IPA3-treated cells were treated with 2.5 μg/ml of PN for 16 h. In some experiments, PN null VICs were embedded in three-dimensional collagen matrices. After polymerization at 37°C for 2 h, the gels were detached from the wells followed by addition of 1 ml of the medium. The potential of embedded cells to contract (compact) the gel lattices was quantified by measuring the decrease in gel diameter over a 24- to 48-h period and expressed as % area of gel contracted.

### Transwell Migration Assays

Transwell migration assays were done using 8-μm-pore-size bottom filter chambers (Corning). Cells (5 × 10^4^) were serum starved overnight and seeded in 200 μl of serum-free Medium 199 onto the upper chamber. The lower compartment was filled with Medium 199 supplemented with 10% serum. After 8 h at 37°C, cells remaining on the upper surface of the filter were wiped off with a cotton swab, and the cells that had migrated on the lower surface of the filter were fixed, stained with DAPI, and counted in 10 microscopic fields.

### Statistical Analysis

Each experiment was repeated three times for each set of fibroblasts, and results were pooled for statistical analysis. Western blot analyses, mRNA analyses, migration, and collagen gel contraction experiments for each separate experiment were repeated between three and four times, depending upon the experiment. Data are expressed as ± SD, or ± SE. The Student’s two tailed *t*-test (Microsoft Excel software) was used for comparison between two groups. Statistical analysis of the Western blots was done using *t-*test with Mann–Whitney modification or analysis of variance (ANOVA) as applicable. When analysis included more than two groups, one-way analysis of variance was used. *P*-values (*p*) ≤ 0.05 were considered statistically significant.

## Results

We have previously shown (Butcher et al., 2007b; Norris et al., 2008b; Markwald et al., 2010; 2019) that prior to ED 17.5, valve progenitor cells are derived by the transformation of endocardial endothelium into free cells that migrate randomly within a hyaluronan-rich, extracellular matrix to form “cushions” that project into the lumen of the heart at the inlet and outlet of the primitive ventricles (Ghatak et al., 2014). During their migration, they express periostin (PN) and differentiate into valve interstitial fibroblasts. At ED 17.5, the mesenchymal-like, valve progenitor cells begin a process of progressively assembling at the base of the primordium (nearest the myocardium) into aligned cell layers or zones separated by linear arrays of condensed or compacted collagenous matrix (Kruithof et al., 2007; Snider et al., 2008). This process continues after birth into neonatal and early adult life (weeks 8–10 in mice) until the entire leaflet is fully compacted or condensed into a characteristic, zonal histoarchitecture that is biomechanically mature (Butcher et al., 2007a; Markwald et al., 2019). Thus, “compaction” is a dynamic, progressive, and fundamental, morphogenetic event in valvulogenesis. What drives compaction is an important question that has clinical relevance to both pediatric and adult heart valve diseases (e.g., mitral prolapse) (Levine et al., 2015). Because periostin expression correlates temporally and spatially with compaction (including the alignment of collagen) during valvulogenesis (Lindsley et al., 2005; Norris et al., 2008a; Snider et al., 2008), and because FLNA is highly expressed during valvulogenesis (Sauls et al., 2012), we propose in this study that periostin signaling may coordinate with FLNA to drive valve morphogenetic compaction. The rationale for this hypothesis is twofold: (i) Pak1 is a downstream target of PN/Integrin β1 signaling (Ghatak et al., 2019), and (ii) FLNA is essential in actin cytoskeletal assembly mediated by Pak1 (Vadlamudi et al., 2002), Also, the similarity in valve phenotypes seen in fetal periostin and FLNA null mice (Norris et al., 2008b) further added to this suggestion and led us to propose that an interaction between PN and FLNA may be required to remodel the valve primordia during fetal and early postnatal life into mature leaflets.

### Periostin Regulates Filamin-A Phosphorylation

**FIGURE 1:**
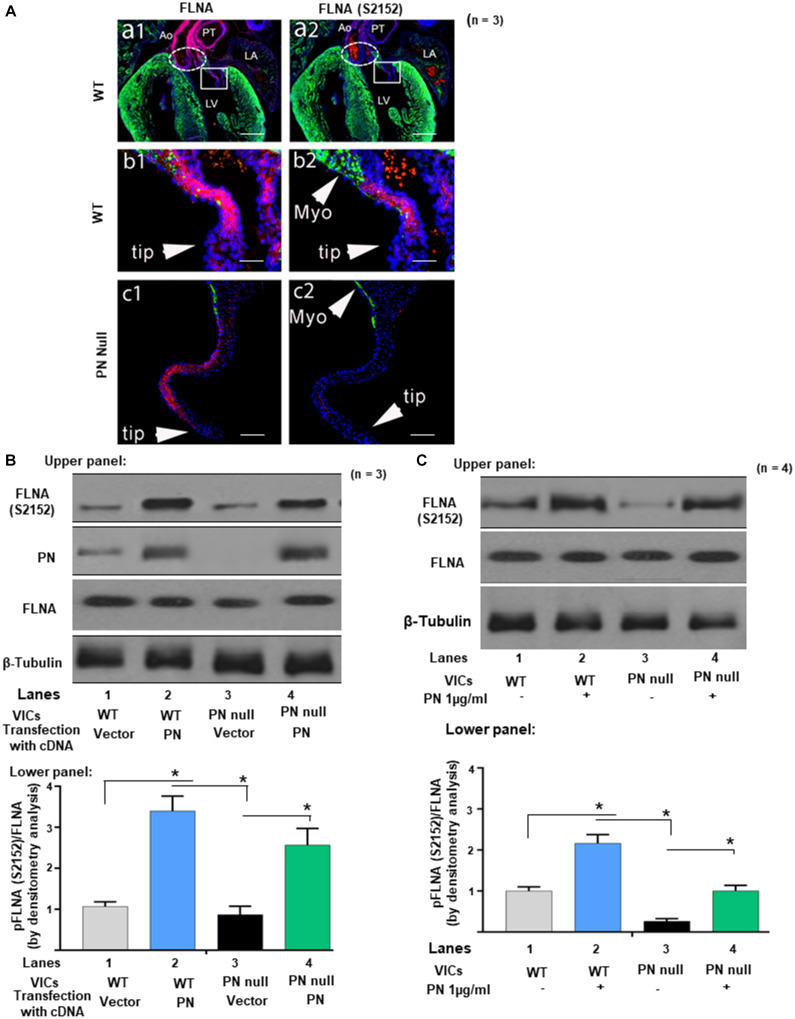
Protein periostin (PN) promotes phosphorylation of filamin A (FLNA) in mitral valve (MV) sections and in valve interstitial cell (VIC) *ex vivo* cultures. **(A)** Labels: The elliptic boxes (a1, a2), indicate the aortic valves. The boxes on a1 and a2 indicate the regions of the mitral valve that are shown at higher magnification on the corresponding b1 and b2. LV, left ventricle; LA, left atria; Myo, myocardium; Ao, Aorta; PT, pulmonary trunk. Green color represents immunostaining for myosin heavy chain (MF20). Blue color represents the nuclear staining Hoechst 33342. Contiguous sections from comparable regions from the mitral valve cushions of the same litter of e17.5 WT (b1, b2) or PN null mice (c1, c2) were used for immunostaining with FLNA and phospho-filamin A [p-FLNA (S2152)]. The p-FLNA (S2152) antibody used gave a clear, strong signal in WT mitral valve cushions at e17.5, which correlated closely with the expression of total filamin A (FLNA). In contrast, PN null valves showed significantly decreased p-FLNA. **(B)** Upper panel: Cell lysates from VICs from e17.5 days were isolated from wild type (WT), and from PN null mice that were transiently transfected with lentivirus encoded with PN cDNA (control, empty vector). They were separated by SDS-PAGE and analyzed by Western blotting (WB) with anti-PN, anti-p-FLNA (S2152), anti-total-FLNA (FLNA), and anti-β-tubulin antibodies. Lower panel: Bars represent densitometric ratios of p-FLNA (S2152)/total FLNA. **(C)** Upper panel: Cell lysates from the VICs that were isolated from WT, and from PN null mice, were serum starved for 24 h, treated with PN (1 μg/ml) for 1 h, and analyzed by WB with anti-p-FLNA (S2152) and anti-total-FLNA antibodies. Lower panel: Bars represent densitometric ratios of p-FLNA (S2152)/Total FLNA. Data in “A” is a representative of three experiments. Values in lower panels of **(B,C)** represent means ± SD; *n* = 3–6; *p < 0.05 for experimental groups compared with respective control groups. Scale bars in panel **(A)** 100 μm. All WBs are representative of three independent experiments.

As a first step in testing this hypothesis, we sought to determine the distribution of FLNA and whether it was phosphorylated in developing wild-type valves at ED 17.5, the time at which compaction normally has begun. Results were then compared to PN null mitral valve leaflets at the same time period, although the PN null phenotype differed from wild-type hearts in shape, size and morphology (Snider et al., 2008; Norris et al., 2009b). Figure 1, panels a1 and a2, are low magnifications of a frontally sectioned wild-type heart at ED 17.5 that shows both the inlet (AV) and outlet valves. Even at low magnification, it can be seen that most of the FLNA protein expressed in the heart at this time point is localized to the valves (Norris et al., 2010).

The two great arteries that emerge from the arteriole pole of the WT heart are also intensely stained for FLNA (red/pink), but they are extracardiac in origin being derived from neural crest cells, not cushion prevalvular cells (Snider et al., 2009). As seen in the upper right panel (Figure 1A, panel a2), phosphorylated FLNA is also detectable in the WT valves but not elsewhere in the heart. At higher magnification (Figure 1A, panels b1 and b2) expression of both FLNA and phosphorylated FLNA in the left (mitral) AV valve (the focus of this study) is primarily localized to the base of the valve (the area closest to the myocardium). This is the same area where compaction is normally initiated and where periostin expression is high (Norris et al., 2008b; Snider et al., 2008). FLNA protein is also expressed on some of the non-compacted prevalvular cells located in the, bulbous valve tips (however, few, if any, of these WT FLNA-positive “tip” cells expressed detectable phosphorylated FLNA, which correlates with the low expression of PN in noncompacted regions of valve primordia) (Norris et al., 2008b, 2009a). In the complete absence of periostin, the mitral valve primordium appears abnormally elongated along the proximal–distal axis and noncompacted [Figure 1A, panels c1, c2; see also (Norris et al., 2008b; Snider et al., 2008)]. PN null interstitial cells express FLNA, but phosphorylated FLNA cannot be detected (Figure 1A, panel c1 and c2). We did not expect to see all null valve cells to express FLNA or pFLNA as differentiation into a fibrogenic lineage is delayed or misdirected into a myocardial cell lineage in the absence of periostin (Norris et al., 2008b; Snider et al., 2008). These observations suggest that periostin is not obligatorily required for synthesis of FLNA by WT or all null valve progenitor cells but potentially for its activation by phosphorylation.

Experiments designed to more directly test whether PN signaling phosphorylates FLNA are shown in Figures 1B,C using VICs that were isolated from PN WT mitral valves at early postnatal period when PN expression normally peaks (Snider et al., 2008), and compaction is nearing completion. Results were compared to those obtained from VICs isolated from periostin-deficient (null) hearts. Western blots show that PN null VICs treated with a vector control also have minimal p-FLNA [Figures 1B,C, lanes 3 compared with lanes 1 (Western Blots in upper panels and densitometric analyses in lower panels)], which is rescued partly when the cells were transduced with PN cDNA (Figure 1B, lane 4 compared with lane 3), or by exogenous treatment with PN (1 μg/ml) (Figure 1C, lane 4 compared with lane 3). The findings in Figures 1A–C are consistent with our hypothesis that phosphorylation of FLNA is downstream of PN signaling and specifically confirm the role of PN in the development of AV mesenchymal (prevalvular) cushion tissue. How PN regulates phosphorylation of FLNA is addressed in the following experiments.

### Inhibition of PN-β1 Integrin-Mediated Cdc42/Rac1 Signaling Blocks Pak Activation and Actin Cytoskeletal Assembly

**FIGURE 2:**
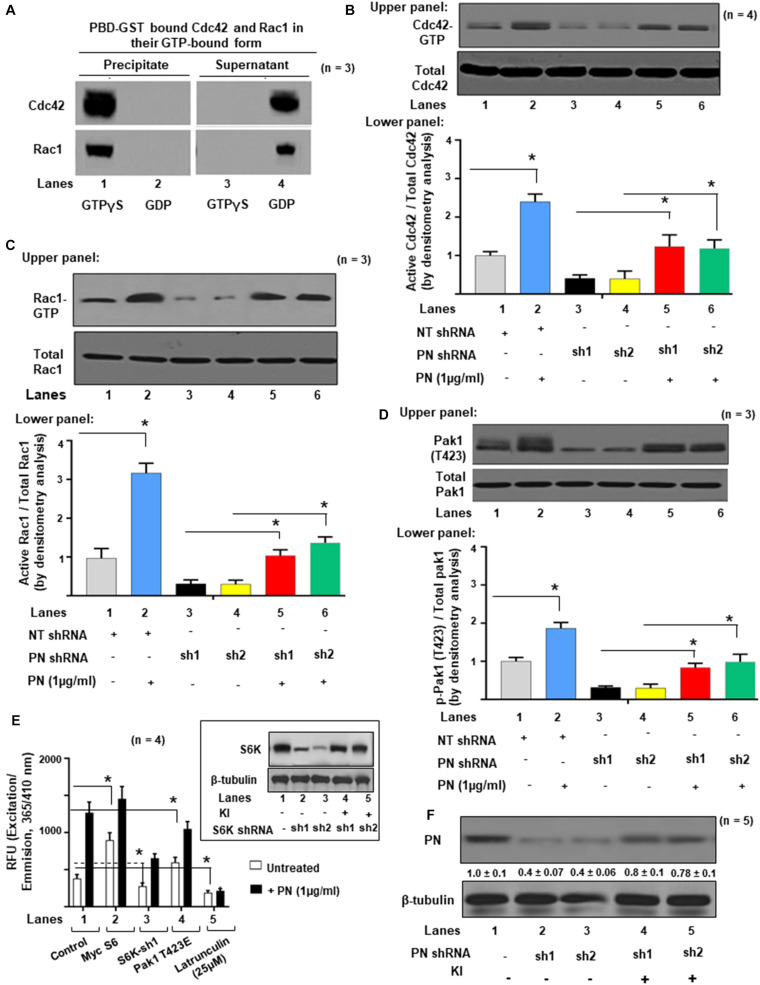
Activations of Cdc42 and Rac1 are associated with Pak1 activation and actin cytoskeletal assembly in VIC *ex vivo* cultures. **(A)** Specific interactions of active Rac1-GTP or Cdc42-GTP with GST-fusion protein of GTPase-binding domain (PBD-GST) of human PAK1 wild type (WT, full length) were measured. VIC lysates were incubated with 100 μM GTPγS or GDP and clarified before precipitation on PBD-GST beads. Supernatants (S) were separated from bead pellets (P) by centrifugation and analyzed by SDS-PAGE using the indicated antibodies. **(B)** Upper panel: Transduced VICs with the indicated shRNAs were serum starved for 16 h and incubated without or with 1 μg/ml PN at 37°C for 1 h. Cdc42-GTP was pulled-down on GTP-loaded PBD-GST beads. The relative amounts of the activation of small GTPases were quantified by normalizing the amount of bound Cdc42 to total Cdc42 in the lysates. Lower panel: Bars represent densitometric ratios of active Cdc42/total Cdc42 in the lysates. **(C)** Upper panel: Transduced VICs with the indicated shRNAs were serum starved for 16 h and incubated without or with 1 μg/ml of PN at 37°C for 1 h. Rac1-GTPs were pulled-down on GTP-loaded PBD-GST beads. The relative amounts of the activation of small GTPases were quantified by normalizing the ratios of the amount of bound Rac1/total Rac1 in the lysates. Lower panel: Bars represent densitometric ratios of active Rac1/total Rac1 in the lysates. **(D)** Upper panel: Pak1 activation was tested by immunoprecipitation with anti-Pak and Western blotting with anti-p-Pak1 [Thr (T) 423]. The total amounts of p-Pak1 in the Pak1 IPs were determined by normalizing the amounts to total Pak1 by densitometry. Lower panel: Bars represent densitometric ratios of p-Pak1 (T423)/total Pak1 in the lysates. **(E)** VICs were transfected with lentivirus encoding NT shRNA, or S6K shRNA, or transfected with Myc-S6 cDNA, and Pak1 T423E, respectively. Then the transfected cells were treated with or without 1 μg/ml PN for 30 min and permeabilized. Fluorescence of pyrene-conjugated actin was measured with a fluorescence spectrophotometer using an actin polymerization kit from cytoskeleton.com. The fluorescence was plotted in samples treated with (black bars) or without (white bars) PN. Inset: Validation was done for the two S6K shRNA used in the experiments in inset of **(E)**. For a detailed description, see the “Experimental Procedure” section. Target proteins were analyzed by WB analyses (β-tubulin was used as an internal control). **(F)** Validation was done for the PN shRNAs used in the experiments of **(B)**. Two shRNAs (shRNA1 and shRNA2) were used for the PN protein. For a detailed description, see the “Materials and Methods” section. Both shRNAs effectively silenced PN gene expression by promoting degradation of their mRNAs. PN shRNA resistant knock-in constructs (labeled KI) were designed to rescue (override) PN-silencing phenotypes. Target proteins were analyzed by WB analyses. β-tubulin was used as an internal control. Results reported in bar graphs in panels **(B–D)** are presented as means ± SD of three separate experiments (*p ≤ 0.01). Western blot data are representative of ≥3 independent experiments. In panel **(F)**, the values were determined by densitometry, and the values derived from control cells (either NT shRNA or untreated cells) were designated as 1.0. Error bars are reported as means ± SD of ≥3 separate experiments (*p ≤ 0.05).

The similarity in valve phenotype of PN and FLNA knockouts (Norris et al., 2008b) is consistent with a regulatory interaction between PN and FLNA in mediating valve matrix assembly (Sauls et al., 2015). Because Paks are targets of small GTPases like Cdc42 and Rac1 (Manser et al., 1994), and since FLNA crosslinks with actin filaments and interacts with Pak1 (Vadlamudi et al., 2002), we investigated whether PN-mediated morphologic changes in valve remodeling correlate with changes in the stimulation of active GTP-bound GTPases, specifically Cdc42 and Rac1. The baseline levels of expression of Cdc42-GTP and Rac1-GTP in neonatal VICs were studied using a PAK-1-binding domain-GST (PBD-GST) pull-down method. The PBD-GST beads were used to entrap Cdc42 and Rac1 in their GTP-bound form from the detergent-soluble fraction of VIC lysates. In the presence of exogenously added GTPγS, full activations of Cdc42 and Rac1 were obtained (Figure 2A, lane 1). No residual Cdc42 or Rac1 was detected in the supernatants (Figure 2A, lane 3). In the presence of an excess of GDP, Cdc42 and Rac1 were maintained in an inactive state, were unable to bind to PBD-GST beads (Figure 2A, lane 2), and remained in the supernatants (Figure 2A, lane 4). These results validated the specificity of PBD-GST beads for the detection of Cdc42-GTP and Rac1-GTP.

The experiment in Figure 2B tested whether small G protein activation was necessary for Pak1 activation as indicated by phosphorylation at T423. VIC cultures were treated with 1 μg/ml of PN for 1 h or transfected with either Control (Cont.) shRNA or PN shRNA for 4 h and then treated with 1 μg/ml PN for 1 h. The levels of activated Cdc42-GTP and of Rac1-GTP were significantly decreased in VICs when they were previously transfected with two different PN shRNAs and compared with non-targeted shRNA (NTsh, control shRNA) transfected cells [Figures 2B,C (Western Blots in upper panel and densitometric analyses in lower panels), lanes 3, 4 compared with lane 1]. Similarly, PN shRNA inhibited the phosphorylation of Pak1 from the basal level [Figure 2D (Western Blots in upper panel and densitometric analyses in lower panels), lanes 3, 4 compared with lane 1]. Activation of Cdc42, Rac1, and Pak1 were partly rescued by treating these cells with PN (Figures 2B–D, lanes 5, 6 compared with lanes 3 and 4). Thus, a blockage of p-Pak1 activity correlates with a reduction in Cdc42-GTP or Rac1, which can be partially reversed by exogenous treatment with PN (Figures 2B–D), suggesting that full activation of both Cdc42 and Rac1 is required for stimulation of p-PAK1 activity by PN. Figure 2F validates the effectiveness of the two shRNAs designed for PN. Both shRNAs effectively silenced PN gene expression by promoting degradation of their transcription and subsequent translation (Figure 2F, lanes 2 and 3 compared with lane 1). PN shRNA-resistant constructs (labeled KI) that were designed to rescue (override) PN silencing reversed the phenotypes (Figure 2F, lanes 4 and 5 compared with lanes 2 and 3) as in our previous study (Ghatak et al., 2017a, b).

We previously showed that a PN/β1 integrin interaction stimulated a focal adhesion kinase (FAK) (Y397)/(PI3K)/Akt1 pathway that linked mTOR/p70 ribosomal protein S6 kinase (S6K) to a Pak1-mediated reorganization of cytoskeletal actin in fetal VICs (Ghatak et al., 2019). To determine if PN has a role in promoting S6K stimulation of Pak1 kinase activity (independent of its role in activating Rac1 and/or Cdc42), we overexpressed S6K, or downregulated S6K by specific shRNA and then treated these transfected cultures with or without PN for 30 min. The effects of knocking down S6K in the presence or absence of PN were tested using a Pyrene-actin polymerization assay. First, actin polymerization was stimulated by treatment with PN for 30 min (Figure 2E, black bar of lane 1 compared with white bar of lane 1). Overexpression of S6K has considerable effect on actin polymerization as measured by fluorescence signals from pyrene G-actinin VICs at time 0 min (Figure 2E, white bars of lane 2 compared with lane 1). Specifically, actin polymerization stimulated by S6K was enhanced by treatment with PN for 30 min (Figure 2E, black bar of lane 2 compared with white bar of lane 2). Blocking S6K with S6K shRNA *partly* inhibited both preexisting polymerized actin and the early movements of G actin into F-actin after stimulation by PN for 30 min (Figure 2E, black and white bars of lane 3 compared with lane 1). Validations of S6K shRNAs on the expression of S6K are shown in the inset of Figure 2E. S6K shRNA-resistant constructs (labeled KI) that were designed to rescue S6K silencing reversed the phenotypes (Figure 2E inset, lanes 4 and 5 compared with lanes 2 and 3) (Ghatak et al., 2017a, b). Knocking down S6K did not suppress all of PN-stimulated actin polymerization (Figure 2E, black bar of lane 3 compared with black bar of lane 1). The residual stimulation of actin polymerization and Pak1 kinase activity after downregulation of S6K by PN (Figure 2E, black bar of lane 3 compared with black bar of lane 1) suggests that in addition to PN/S6K, other signaling cascades, including the small GTPases (Cdc42 and Rac1) may also mediate the potential of PN to stimulate actin polymerization and Pak1 kinase activity. To determine whether the Pak1 pathway is required for actin polymerization in wild-type VICs, we examined the effect of a constitutively active Pak1 (Pak1 T423E) expression vector on the actin polymerization by PN signals. Actin polymerization was induced in wild-type VICs expressing the constitutively active Pak1 compared with the control (Figure 2E, white bars of lane 4 compared with white bar of lane 1), and combined treatment of Pak1 T423E with PN further stimulated actin polymerization (Figure 2E, black bars of lane 4 compared with white bar of lane 4). Together, these results indicate that the actin polymerization is mediated by PN, and it requires S6k/Pak1 signaling. Moreover, PN/S6K signaling for actin polymerization was dependent on the integrity of the cytoskeleton because disruption of the actin cytoskeleton assembly by latrunculin considerably reduced both the extent of basal and PN-enhanced actin polymerization (Figure 2E, black and white bars of lane 5 compared with black and white bars of lane 1).

**FIGURE 3:**
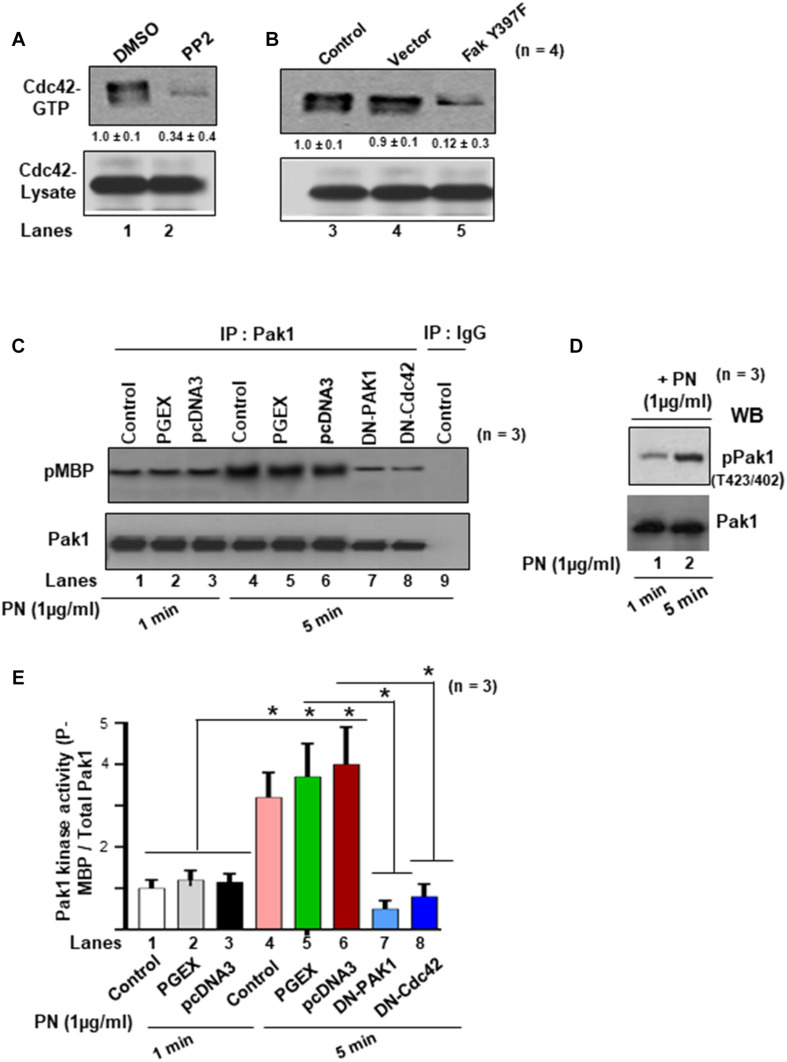
Activations of Cdc42 and Rac1 are associated with PN, which induced both the autophosphorylation and the kinase activity of Pak1 in the VIC *ex vivo* cultures. **(A)** Serum-deprived cells were cultured in the presence of either DMSO or Src inhibitor PP2 (5 μM). Pull-down assays for active Cdc42 were done using GTP-loaded PAK-1-binding domain (PBD)-GST beads. **(B)** Cells were either uninfected (control), or infected with lentivirus-expressing vector, or Fak (Y397F) and analyzed for active Cdc42 as in Figure 2B. **(C)** VICs were either transfected with DN Pak1 or DN Cdc42 constructs for 48 h. Cells were treated with 1 μg/ml PN for 1 or 5 min at 37°C, and a nonradioactive kinase assay was done using the traditional in-gel phosphorylation assay. For details of this experiment see the “Materials and Methods” section. Pak1 kinase activity was evaluated by phosphorylation of substrate MBP (pMBP). The amount of immunoprecipitated PAK was controlled by anti-PAK1 immunoblotting. Isotype control IgG was used as experiment control. **(D)** Autophosphorylation of PAK (pPAK) was measured after 1- and 5-min treatments with PN [substrate 1 μg of myelin basic protein (MBP)] and was visualized as a 65-kDa phosphoprotein and detected by direct immunoblotting with anti-phospho PAK1/2 Thr423/Thr402. Results are representative of two separate experiments. **(E)** PAK activation was evaluated as *in vitro* kinase activity toward MBP. Quantification of PAK activity from the experiment of **(C)** was done by densitometry analysis on ratio of expression of pMBP and total Pak1. In panel **(A)**, the values were determined by densitometry, and the values derived from control cells (either NT shRNA or untreated cells) were designated as 1.0. Results reported in bar graphs in panel **(E)** are presented as means ± SD of ≥3 separate experiments (*p ≤ 0.05). Western blot data are representative of ≥3 independent experiments.

Previously, it was shown that FAK regulates cytoskeletal reorganization in response to many integrins (Cary and Guan, 1999) and that FAK–Src complexes promote the activation of Rac1 and Cdc42, two GTPases involved in the remodeling of the actin cytoskeletal network (Hernandez et al., 2018). However, we found that only CDC42 was effective in inducing Pak1 kinase in cells that had been previously stimulated to enhance periostin signaling pathways (Ghatak et al., 2019). A recent study also demonstrated that activated GTP forms of Cdc42 and Rac1 have different cellular localizations: Cdc42 associates with actin cytoskeleton, whereas Rac localizes to the plasma membrane during cellular migration/invasion (Azim et al., 2000). Therefore, we sought to determine whether PN/α5β1 integrin signaling through FAK/Src promoted the activation of Pak in VICs and, if so, whether this activation also correlated with activation of the Cdc42-GTPases. To do so, we tested whether inhibiting Src or FAK leads to reduced levels of active Cdc42 in wild-type VICs that express PN and α5β1 integrin (Ghatak et al., 2014, 2019). WT-VICs treated with the Src inhibitor PP2 reduced by threefold the level of active Cdc42 compared with DMSO-treated control cells (Figure 3A, lane 2 compared with lane 1). Similarly, WT-VICs that were transfected with inhibitory Fak Y397F showed a five- to eightfold reduction in levels of active Cdc42 compared with vector control cells (Figure 3B, lane 5 compared with lane 4). Thus, a role for both Src and FAK are indicated in the stimulation of Cdc42 activity by PN in WT VICs, suggesting a model wherein PN/α5β signaling promotes Pak1 activation through a pathway that involves a FAK/Src/Cdc42 pathway. To further test this model, we quantified the activation of PAK following PN stimulation in VICs using an *in vitro* phosphorylation assay to analyze Pak autophosphorylation and VICs Pak-kinase activity toward purified myelin basic protein (MBP) as an exogenous substrate. Quantification of phospho (p) MBP to total Pak by densitometry analysis showed that MBP phosphorylation increased from 1 to 5 min (Figures 3C,E). PN induced an increase in MBP phosphorylation after 5 min of stimulation, whereas dominant negative (DN)-Pak1 and DN-Cdc42 inhibited phosphorylation of MBP (Figures 3C,E, lanes 7 and 8 compared with lanes 5 and 6, respectively). Anti-Pak1 immunoblots showed that the amounts of immunoprecipitated Pak1 were equal. Isotype control IgG was used as control (Figure 3C, lane 9). The occurrence of Pak autophosphorylation in VICs stimulated by PN for 1 or 5 min was confirmed by direct immunoblot with anti-pPak1/2 (Thr423/Thr402) antibody (Figure 3D, lanes 1 and 2). Thus, these results further support the model that PN induces an activation of Pak1 in VICs through FAK/Src by a mechanism that correlates with activation of the Cdc42-GTPases.

**FIGURE 4:**
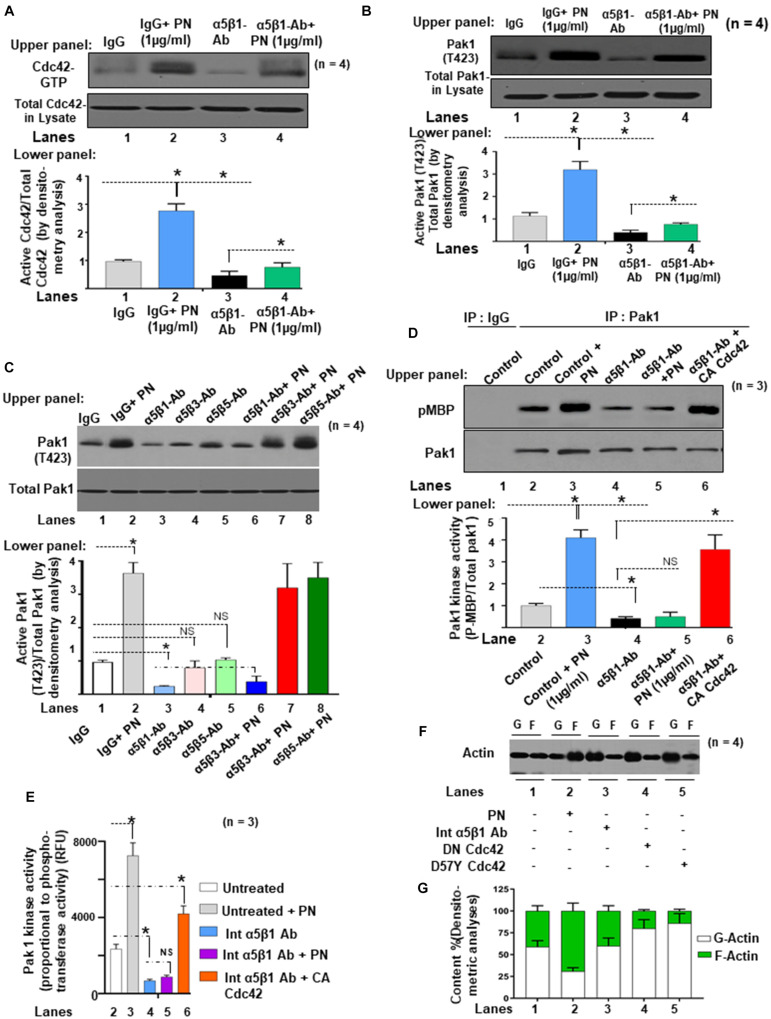
PN/integrin β1-regulated Cdc42 activation is associated with stimulation of both the autophosphorylation and the kinase activity of Pak1 and actin cytoskeletal assembly in the VIC *ex vivo* cultures isolated from ED 17.5 mice mitral valves. **(A)** Upper panel: Cell lysates were harvested from growth-arrested VIC cultures that were either treated with or without 1 μg/ml of PN for 1 h (in serum-starved condition), or first treated with 5 μg/ml of both α5 and β1 integrin blocking antibodies for 2 h and then treated with 1 μg/ml of PN for 1 h. Cell lysates were prepared, and Cdc42-GTP was pulled-down from the lysates on GTP-loaded PBD-GST beads. The relative amounts of the activation of Cdc42 were quantified by normalizing the amount of bound Cdc42 to that of total Cdc42 in the lysates. Lower panel: Bars represent densitometric ratios of active Cdc42/total Cdc42 in the lysates. **(B)** Upper panel: Pak1 activation was tested by immunoprecipitation with anti-Pak and Western blotting with anti-p-Pak1 [Thr (T) 423]. The total amounts of p-Pak1 in the Pak1 IPs were determined by normalizing the amounts to total Pak1 by densitometry. Lower panel: Bars represent densitometric ratios of phospho p-Pak1 (T423)/total Pak1 in the lysates. **(C)** Upper panel: VICs were either treated with or without 5 μg/ml of both α5 and β1 integrin blocking antibodies for 2 h, or first treated with these blocking antibodies and then transfected with constitutively active (CA) Cdc42 cDNA for 48 h. PAK was immunoprecipitated from these treated/transfected VICs that were stimulated with 1 μg/ml PN for 5 min at 37°C, and a nonradioactive kinase assay was done using the traditional in-gel phosphorylation assay. For details of this experiment, see the “Materials and Methods” section. Pak1 kinase activities were evaluated by phosphorylation of substrate MBP (pMBP). The amounts of immunoprecipitated PAK were controlled by anti-PAK1 immunoblotting. Isotype control IgG was used as control. Lower panel: Bars represent densitometric ratios of active Cdc42/total Cdc42 in the lysates. **(D)** Upper panel: VICs were transfected with indicated lentivirus-expressing vectors. After 72 h, they were treated with and without PN for the indicated times. PAK was immunoprecipitated from platelets stimulated at indicated times at 37°C and incubated with 1 μg of MBP in the presence of 20 μM MgCl_2_-ATP and 0.185 MBq (5 μCi) γ[32P]-ATP (for details, see the “Materials and Methods” section). Autophosphorylated PAK was visualized as a 65-kDa phosphoprotein, and its kinase activity was evaluated by phosphorylation of MBP (pMBP). The amount of immunoprecipitated PAK was controlled by anti-PAK1 immunoblotting. Isotype control IgG was used as experiment control. Lower panel: PAK activation from the experiment of **(D)** was evaluated as *in vitro* kinase activity toward MBP, and quantification of PAK activity was done by densitometry analysis on ratio of expression of pMBP and total Pak1. **(E)** *In vitro* Pak1 kinase assays were further confirmed by nonradioactive assay kit (ab138879, Abcam), which is based on the monitoring of ADP formation (see “Materials and Methods” section for details). Data are presented as fold increase in Pak1 kinase activity with respect to control. **(F,G)** The effects of dominant negative mutants of N17 Cdc42 (DN Cdc42) and D57Y Cdc42 as well as the impact of blocking antibody for α5 and β1 integrin on actin dynamics were assayed. VICs were transfected with the indicated lentiviral constructs and incubated for 1 h with 10 μM G-actin, or in the presence or absence of 1 μg/ml of PN for 1 h. Lysates were assayed to determine actin dynamics by measurements of changes in the ratios **(G)** of F-actin/G-actin by immunoblot densitometry **(F)**. The data in experiments in panels **(A–G)** are representative of ≥3 independent experiments. The values derived **(A–G)** from control cells were designated as 1.0. Error bars are reported as means ± SD of ≥3 separate experiments. *p < 0.05 was considered significant.

Next, we determined whether PN could stimulate an interaction between Pak1 with Cdc42 through binding to its canonical integrin receptor (α5β1 integrin) using a Pak-1/PBD-GST pull-down assay in the presence or absence of blocking antibody for α5β1 integrin. Western blots confirmed in VIC controls that PN stimulated the activation of Cdc42 (Cdc42-GTP) and p-Pak1 (T423) above their base line levels [Figures 4A,B, lanes 2 compared with lanes 1 (Western blots in upper panel and densitometric analyses in lower panels)]. However, treatment of these WT cells with a blocking antibody to α5β1 integrin strongly suppressed the binding of GTP-activated Cdc42 to p-Pak1 (Figures 4A,B, lanes 3 compared with lanes 1, upper and lower panels), which could be overridden in a very minor level by the addition of PN (Figures 4A,B, lanes 4 compared with lanes 2). The residual minor reversal of the inhibitory effect of α5β1 integrin-blocking antibody on Cdc42 and Pak1 activation even after stimulation of PN (Figures 4A,B, lanes 4 compared with lanes 3) suggests that other signaling cascades involving PN/α5β3 or PN/α5β5 integrin signaling may also regulate Pak1 kinase activation. However, treatment of these WT cells with a blocking antibody to either α5β1, or α5β3, or α5β5 in the same experiment showed that only blocking α5β1 downregulated p-Pak1 significantly (Figure 4C, lane 3 compared with lane 1 versus lanes 4 and 5 compared with lane 1). The inhibitory effect of blocking α5β1 was only overridden in a very minor level by the addition of PN, and blockage with α5β3 or α5β5 showed no effect on activation of Pak1 (Figure 4C). This provides evidence that PN/α5β1 signaling has the vital role in the activation of Pak1 kinase.

Next, we measured the impact of blocking α5β1 expression on Pak1 kinase activity by quantifying phosphorylation of MBP (Figure 4D), and by measuring Pak1 kinase activity as assessed by quantifying phosphotransferase activity (Figure 4E). Importantly, PN treatment to VICs promoted the Pak1 kinase activity in controls [Figure 4D (both upper and lower panels), Figure 4E, lanes 3 compared with lanes 2] but had little or no effect in the presence of an integrin-blocking antibody [Figure 4D (both upper and lower panels), Figure 4E, lanes 5 compared with lanes 4], suggesting that PN/α5β1 integrin signaling is necessary for activation of Pak1 kinase activity. Blocking α5β1 integrin reduces the basal Pak1 kinase activity [Figure 4D (both upper and lower panels), Figure 4E, lane 4 compared with lane 1]. While the inhibition of Pak1 phosphorylation and kinase activity resulting from the presence of the α5β1 integrin antibody was not reversed by exogenous addition of PN, it was appreciably reversed by transfection of vectors that constitutively activated Cdc42 (CA Cdc42) [Figure 4D (both upper and lower panels), Figure 4E, lane 6 compared with lanes]. The residual minor inhibition of Pak1 kinase by α5β1 integrin blockage even after overexpression of CA Cdc42 [Figure 4D (both upper and lower panels), Figure 4E, lane 6 compared with lane 4], suggests that other signaling cascades, in addition to PN/α5β1 integrin, may also regulate Pak1 kinase through Cdc42.

To validate that the interaction of Cdc42 and Pak1 promoted actin cytoskeleton assembly, we measured the content of G-actin and F-actin in fetal VICs transfected with mutated plasmids that knocked down expression of Pak1 and Cdc42 (DN-Pak1 and DN-D57Y Cdc42), or the fetal VICs were treated with blocking α5β1 integrin antibody in the presence and absence of added PN (Figures 4F,G). The polymerized F-actin to G-actin ratios shown in Figures 4F,G indicate that the potential of PN to promote actin reorganization (Figures 4F,G, lane 2 compared with lane 1) was impaired in the presence of the blocking α5β1 Integrin antibody (lane 3 compared with lane 1), or by DN Cdc42 or Pak1 inactive mutants (lanes 4, 5 compared with lane 1). Furthermore, the addition of PN could not restore the blockade of G-actin polymerization into F-actin by blocking α5β1 Integrin antibody, or by DN Cdc42 or DN Pak1 (data not shown). These findings indicate that normally, the binding of PN to α5β1 activates signaling cascades that are mediated by Cdc42 and enhances Pak1 kinase activity. This provides a probable mechanism by which PN signaling can promote F-actin polymerization and cytoskeletal reorganization through Cdc42-Pak1 signaling.

### FLNA Is a Cdc42-Pak1-Binding Protein

**FIGURE 5:**
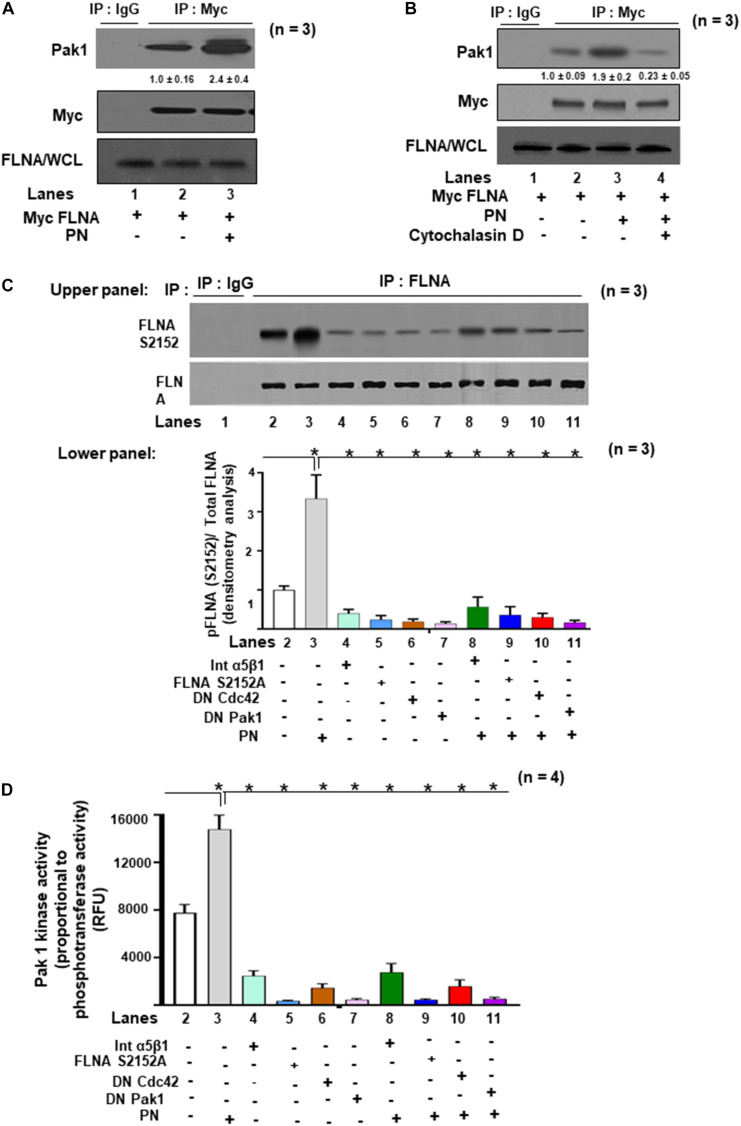
The effects of PN/integrin β1 on association of Pak1 with FLNA, a substrate for Pak1 kinase activity in VIC lysates. **(A,B)** VICs were transfected with lentivirus encoding Myc-FLNA for 48 h and then serum starved for 24 h. They were then either untreated or treated with 1 μg/ml of PN for 1 h. **(A)** Cell lysates were immunoprecipitated without (lane 1, control IgG) or with an antibody against Myc (for FLNA) and immunoblotted with antibodies against Pak1, Myc, or FLNA. **(B)** VICs were pretreated with cytoskeleton disrupter 2 μM cytochalasin D (CytoD) for 30 min and then treated with 1 μg/ml PN for 1 h (lane 4) and compared with VICs treated with PN alone (lane 3). Cell lysates were immunoprecipitated with an antibody against Myc (for FLNA) and immunoblotted with antibodies against Pak1, Myc, or for FLNA in WCL. Lane 1 is for control IgG. **(C)** Upper Panel: VICs were cotransfected with the FLNA S2152A mutant, or DN Pak1 containing Pak1 autoinhibitory domain (residues 83–149), or DN Cdc42, or 5 μg/ml of α5 and β1 integrin blocking antibodies for 2 h. After 48 h, the cells were serum starved, or treated with or without 1 μg/ml of PN as in the experiment in Figure 2G. Pak1 was immunoprecipitated, and a nonradioactive kinase traditional in-gel phosphorylation assay was done using GST fusion proteins of FLNA as substrate. Isotype control IgG was used as control. For details, see the “Materials and Methods” section. Lower Panel: Bars represent densitometric ratios of active phospho pFLNA (S2152)/total FLNA in the lysates. **(D)** *In vitro* Pak1 kinase assays from the experiment in panel **(C)** were further confirmed by assaying using the nonradioactive assay kit (ab138879, Abcam), which is based on the monitoring of ADP formation (see “Materials and Methods” section for details). Data are presented as fold increase in Pak1 kinase activity with respect to control. The data in the experiments in panel **(A–D)** are representative of ≥3 independent experiments. The values in experiments **(A–B)** were determined by densitometry. The values derived from the control cells were designated as 1.0. Error bars are reported as means ± SD of ≥3 separate experiments.

Having found that activated Pak1 can crosslink with actin filaments following PN stimulation (Figures 4F,G) and that PN can also trigger downstream phosphorylation of FLNA S2152 (Figure 1), we investigated whether Pak1 and FLNA can directly interact in VICs after stimulation to induce reorganization of the cytoskeleton. To address this, we overexpressed Myc-tagged-FLNA in VICs. These transfected VICs were then treated with or without 1 μg/ml of PN for 30 min. Figure 5A shows that PN enhanced the association of FLNA and Pak1 (lane 3 compared with lane 2; also see Figure 5B, lane 3 compared with lane 2), as determined by co-immunoprecipitation assays using antibodies against Myc and by Western blot analysis of Myc FLNA immunoprecipitates for Pak1 and Myc. The association of FLNA with Pak1 appeared to depend on the integrity of the cytoskeleton because disruption of the cytoskeleton by cytochalasin D considerably reduced the extent of PN-enhanced Pak1 colocalization with FLNA (Figure 5B, lane 4 compared with lane 3), suggesting that an intact cytoskeletal structure is essential for the PN-mediated interaction of Pak1 and FLNA. As noted above, PN induces cytoskeletal rearrangements through activation of Pak1 (Figure 2E, black bar versus white bar in lane 4), and PN induced the association of FLNA with Pak1 (Figures 5A,B). Moreover, FLNA is essential for the formation of actin cytoskeletal assembly (Vadlamudi et al., 2002). Thus, we examined whether PN stimulates Pak1 kinase activity that phosphorylates endogenous FLNA. To show that FLNA is a direct substrate of Pak1 in response to PN, an *in vitro* kinase assay was performed using purified GST–fusion protein of FLNA residues (2,100–2,550). WT VICs were cotransfected with FLNA S2152A, kinase-dead Pak1 (DN Pak1), or kinase-dead Cdc42 (DN Cdc42), and then serum starved and treated with or without PN or blocking antibody for α5β1 integrin. FLNA was immunoprecipitated, and phosphorylation status was assayed (Figure 5C). Furthermore, we measured Pak1 kinase activity by measuring phosphotransferase activity (Figure 5D). Results indicate that PN added to the culture medium induced both the phosphorylation of FLNA at S2152, and Pak1 kinase activity [Figure 5C (both upper and lower panels), Figure 5D, lane 3 compared with lane 2]. Furthermore, basal FLNA phosphorylation could be reduced significantly by treatment with either α5β1 integrin blocking antibody (1 μg/ml for 2 h), by transfection with FLNA S2152A mutant, by inhibiting Cdc42 activation, or by inhibiting Pak1 kinase activity [Figure 5C (Western blots in upper panel and densitometric analyses in lower panels), Figure 5D, lanes 4–7 compared with lane 2]. Moreover, PN (1 μg/ml) stimulation for (30 min) reversed a very minor level of the inhibitory effect of α5β1 integrin antibody (1 μg/ml for 2 h) by 10–20% (Figures 5C,D, lane 8 compared with lane 4), suggesting that in addition to PN/α5β1 integrin, other signaling cascades such as PI3K and PI3K/Akt-dependent MAPK/Erk may also mediate the potential of PN to stimulate activation of FLNA through Pak1. Intriguingly, FLNA protein has also been confirmed as a substrate for other protein kinases, including MAPK (He et al., 2003) and Akt (Ravid et al., 2008), which are involved in initiating FLNA interaction with cytoskeleton remodeling proteins or integrins, and most of these interactions occur through the C-terminal region of FLNA (Stossel et al., 2001). However, cotransfection with DN Cdc42, DN Pak1, or mutant FLNA S2152A inhibited the potential of PN to induce phosphorylation of FLNA and Pak1 kinase activity in WT-VIC cells (Figures 5C,D, lanes 9–11 compared with lane 3). These findings, when considered together, indicate that PN/α5β1 interaction signaled the downstream phosphorylation of FLNA *in vitro* through Pak1 kinase (Figures 5C,D), findings consistent with *in vivo* imaging *of* FLNA phosphorylation by immunostaining and by Western blotting of E17.5-day PN null fetal hearts (Figures 1A,B). These results indicate that FLNA phosphorylation is a crucial component of PN downstream signaling to regulate valve development.

### Impact of Mutations in FLNA on Mitral Valve Remodeling

**FIGURE 6:**
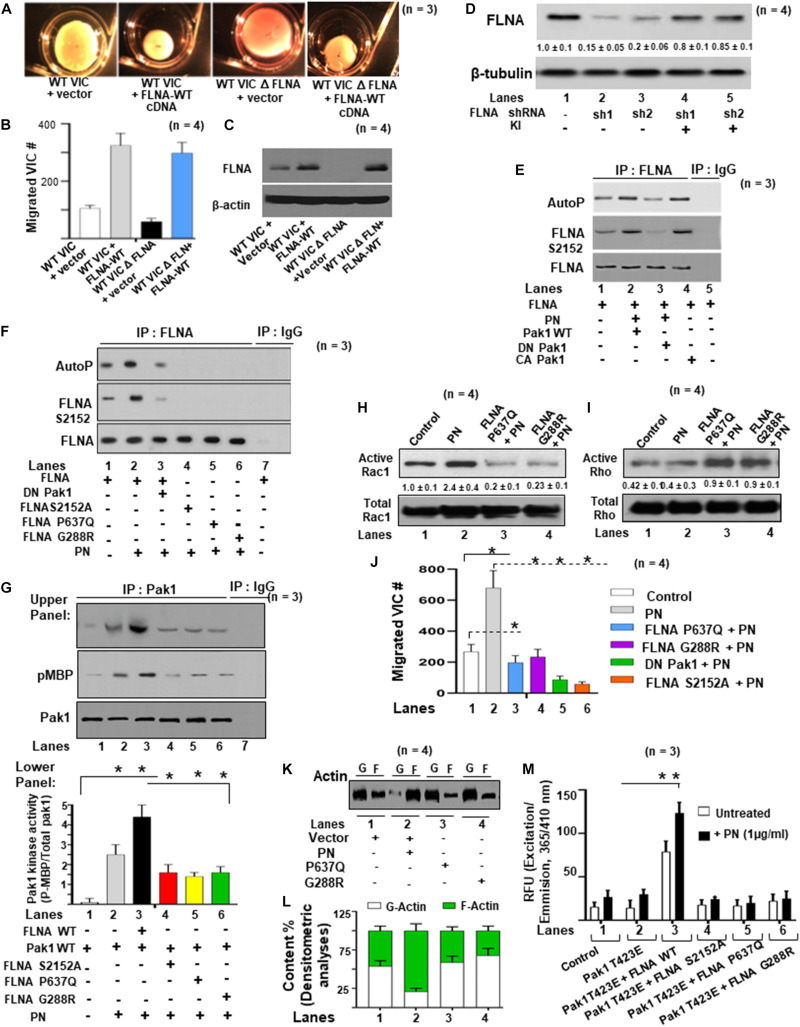
FLNA is a substrate of Pak1, and the effects of FLNA mutations [FLNA (P637Q) and FLNA (G288R)] on PN induced FLNA phosphorylation, on activation of Rac1, and on cell migration are shown. **(A)** VICs were transfected with FLNA shRNA. The stable clones were isolated and designated as FLNA-deficient VICs (VICs Δ FLNA) WT VICs and FLNA Δ VICs were transfected with vector or with wild-type FLNA lentiviral constructs for 72 h before treatment. These transfected VICs were then embedded in three-dimensional collagen matrices to analyze collagen lattice compaction (see the “Materials and Methods” section for details). **(B)** Transwell migration assays are shown for WT VICs and VICs Δ FLNA pre-transfected with the indicated lentiviral constructs. Percentages of cells seeded in the upper chamber of Transwell filters that migrated overnight into the lower chamber are shown. Numbers of migrated cells were counted on six fields from three experiments. Detailed descriptions are in the “Materials and Methods” section. **(C)** Western blot analyses are shown for FLNA and β-actin (internal control) proteins in the lysates from WT VICs and VICs Δ FLNA pretransfected with the indicated lentiviral constructs. **(D)** Validation was done for the FLNA shRNAs used in the experiments of **(A–C)**. Two shRNAs (shRNA1 and shRNA2) were used for the FLNA protein. For a detailed description, see the “Materials and Methods” section. Both shRNAs effectively silenced FLNA gene expression by promoting degradation of their mRNAs. FLNA shRNA resistant knock-in constructs (labeled KI) were designed to rescue (override) FLNA silencing phenotypes. Target proteins were analyzed by WB analyses. β-tubulin was used as an internal control. **(E)** VICs Δ FLNA were transfected with wild-type FLNA plus wild-type Pak1 (Pak1 WT), kinase-dead [dominant negative (DN)] Pak1, or constitutively active (CA) Pak1, and then serum starved, labeled with ^32^P, and treated with or without 1 μg/ml of PN for 30 min. FLNA was immunoprecipitated, and autophosphorylated FLNA was visualized as a 280-kDa phosphoprotein in autoradiography. Phosphorylation of FLNA (S2152) was evaluated in Western blotting using FLNA (S2152)-antibody and total FLNA-antibody. **(F)** FLNA-deficient VICs were cotransfected with lentiviral constructs for DN Pak1, FLNA-WT, FLNA S2152A, FLNA P637Q, or FLNA G288R mutants, serum starved, labeled with ^32^P, and treated with or without PN. The control culture was not treated with PN. FLNA was immunoprecipitated, and autophosphorylated FLNA was visualized as a 280-kDa phosphoprotein in autoradiography. Phosphorylation of FLNA (S2152) was evaluated in Western blotting using FLNA (S2152)-antibody and total FLNA-antibody. **(G)** FLNA-deficient VICs were transfected with Pak1-WT, FLNA-WT, FLNA S2152A, FLNA P637Q, FLNA G288R mutant, or DN Pak1. They were then treated with or without 1 μg/ml PN for 30 min, immunoprecipitated with Pak1, and analyzed for Pak1 activity using myelin basic protein (MBP) as a substrate. Upper panel: Western blotting for indicated pMBP and total Pak1 in Pak1 IP (IgG IP was used as control) and lower panel: Bars represent densitometric ratios of pMBP/total Pak1 in Pak1 IP. **(H,I)** Lysates from VICs transfected with the indicated lentiviral FLNA constructs were processed for immunoprecipitation with GTP-loaded PBD-GST beads. Proteins associated with the beads were then extracted and analyzed by immunoblotting with Rac1 **(H)** and RhoA **(I)** antibodies. The altered proportions of bound Rac1 **(H**) and RhoA **(I)** relative to the control sample are shown. **(J)** Transwell migration assays are shown for VICs pretransfected with the indicated lentiviral constructs, serum starved for 24 h, and then treated with 1 μg/ml of PN for 1 h. Detailed descriptions are in the “Materials and Methods” section. Percentages of cells seeded in the upper chamber of Transwell filters that migrated overnight into the lower chamber are shown. Numbers of migrated cells were counted on six fields from three experiments. **(K,L)** The effects of indicated FLNA mutants on actin dynamics were assayed in FLNA-deficient VICs. VICs were transfected with the indicated lentiviral constructs and incubated for 1 h with 10 μM G-actin, or in the presence or absence of 1 μg/ml of PN for 1 h. Lysates were assayed to determine actin dynamics by measurements of changes in the ratios of F-actin/G-actin by immunoblot **(K)** and densitometry analyses [bar graphs in **(L)**]. **(M)** FLNA-deficient VIC Δ FLNA cells were transfected with lentivirus encoding Pak1T423E followed by cotransfection with FLNA-WT and FLNA mutants (S2152A, P637Q, and G288R), respectively. Then the transfected cells were treated with or without 1 μg/ml of PN for 30 min and permeabilized. Fluorescence of pyrene-conjugated actin was measured with a fluorescence spectrophotometer using an actin polymerization kit from cytoskeleton.com. The fluorescence was plotted in samples treated with (black bars) or without (white bars) PN. The values in each experiment **(D,G,H)** were determined by densitometry. The values derived from control cells were designated as 1.0. Error bars are reported as means ± SD of ≥3 separate experiments. Results in panels **(A–K)** are representative of ≥3 separate experiments. Error bars represent SD. *p < 0.01 was considered significant.

Two-point mutations in the actin-binding regions P637Q and G288R of FLNA were discovered in patients with mitral valve prolapse (Kyndt et al., 1998, 2007; Lardeux et al., 2011; Duval et al., 2014, 2015). Family members with one or more of these mutations had severe forms of X-linked, myxomatous valve degeneration characterized by a progressive loss of matrix compaction and organization that mechanically interfered with valve coaptation and resulted in increased potential for mitral valve prolapse and regurgitation (Duval et al., 2014, 2015). In agreement with the previous studies (Vadlamudi et al., 2002; Hammer et al., 2013), we have shown *in vitro* that FLNA is a substrate for PAK1, and PN is an inducer of Pak1 kinase activity on the phosphorylation of FLNA (Figures 5C,D). To further study the possibility that PN-regulated Pak1 can phosphorylate FLNA *in vivo*, we stably transfected the VICs with our FLNA shRNA construct and isolated an FLNA deleted VIC clone, referred to as FLNA-deficient VICs (VIC Δ FLNA, validation of FLNA shRNA is shown in Figure 6D). These cells do not express detectable levels of FLNA (Figure 6C), and are defective in cell differentiation as measured by a collagen gel compaction assay (Figure 6A) and show reduced migration (Figure 6B). The VIC Δ FLNA cells have this phenotype reversed through the stable expression of wild-type FLNA protein (Figures 6A–C). Data in Figure 6E show that cotransfection of wild-type Pak1, but not DN-Pak1, induced PN-mediated phosphorylation of FLNA in VIC Δ FLNA cells assayed by metabolically labeling with γ^32^P-orthophosphoric acid. Cotransfection of constitutively active Pak1 [CA Pak1 (Pak1T423E)] additionally stimulated autophosphorylation of FLNA (Figure 6E, lane 4 compared with lane 1). These results further confirm that PN induced Pak1-mediated phosphorylation of FLNA in VIC Δ FLNA cells.

Since Pak1 phosphorylation sites were localized in the region of FLNA at the S2152 residue (Vadlamudi et al., 2002), we sought to determine whether mutation of Ser 2152 to alanine, or FLNA P637Q, or G288R mutations affect phosphorylation of FLNA by Pak1. To establish further the role of these FLNA mutants on FLNA phosphorylation by Pak1, transient transfection assays were analyzed in VIC Δ FLNA cells using each of these FLNA mutants as well as FLNA-WT constructs (Figure 6F). These cells do not express detectable quantities of FLNA protein (Figure 6C). Treating VIC Δ FLNA cells transfected with wild-type FLNA and with PN vector induced the FLNA autophosphorylation, and an increase in endogenous FLNA phosphorylation (S2152), but not in the mutant FLNAs (Figure 6F, lanes 4–6 compared with lane 2). However, cotransfection of a DN-Pak1 construct significantly reduced the PN-induced phosphorylation of wild-type FLNA (Figure 6F, lane 3 compared with lane 2). These findings indicate that PN regulated Pak1 kinase activity may stimulate the autophosphorylation of FLNA and phosphorylation of endogenous FLNA through Pak1 both *in vitro* and *in vivo.*

We next examined the impact of FLNA on the stimulation of Pak1 kinase activity in VIC Δ FLNA cells. We found that transient coexpression of FLNA and wild-type Pak1 enhances the kinase activity associated with wild-type Pak1 stimulated by PN [Figure 6G (Western blots in upper panel and densitometric analyses in lower panels), lane 3 compared with lane 2]. As shown in Figure 6G (Western blots in upper panel and densitometric analyses in lower panels, lanes 4–6 compared with lane 3), each of these mutants (FLNA S2152A or P637Q, or G288R) expression failed to increase the Pak1 kinase activity in response to PN signaling. These results suggest that FLNA stimulates Pak1 activity in response to PN.

As noted in previous sections (Figures 4, 5) indicate that PN regulates Pak1 activity to stimulate the phosphorylation of FLNA and that FLNA may have a role in maintaining Pak1 kinase activation. These results further suggest that PN/α5β1 interaction is a physiological regulator of bidirectional interactions between Pak1 and FLNA, and that patients with point mutations within the FLNA protein can interfere with this bidirectional event, possibly contributing to a prolapsed mitral valve phenotype.

Because each of the FLNA mutations (P367Q and G288R) are known to alter the balance between RhoA and Rac1 GTPases (Duval et al., 2014), and inhibit FLNA S2152 phosphorylation (Figure 6F, lanes 5 and 6 compared to lane 2), we examined whether these point mutants affect FLNA functionality including actin nucleation, migration, and differentiation of VICs. Using WT-VICs that were transiently transduced with each of the FLNA (G288R) or FLNA (P637Q) mutants, we observed that active Rac1 was reduced after transduction by more than 70–80% with respect to that for PN-induced VICs (Figure 6H, lanes 3, 4 compared with lane 2). Conversely, RhoA activity was significantly elevated after transfection with FLNA (P637Q) and FLNA (G288R) compared with WT-FLNA-transduced VICs (Figure 6I, lanes 3, 4 compared with lane 2). We then examined whether the decrease in Rac1 activity that followed transfection with FLNA (G288R) and FLNA (P637Q) mutants had any impact on the migratory behavior of VICs. Whereas PN treatment significantly increased migration in nontransfected WT control valve cells (Figure 6J, lane 2 compared with lane 1), transfection with mutant FLNA (G288R or P637Q) abrogated the migratory effect of PN (Figure 6J, lanes 3, 4 compared with lane 2). These results indicate that the G288 and P637 sites of WT FLNA are directly or indirectly related to the potential of periostin signaling to phosphorylate FLNA at the S2152 site (Figure 6F, lanes 5, 6 compared with lane 2), to inhibit Pak1 kinase activity [Figure 6G (Western blots in upper panel and densitometric analyses in lower panels) lanes 5 and 6 compared with lane 3], to inhibit Rac1 and activate Rho (Figures 6H–I, lanes 3 and 4 compared with lane 2), to inhibit VIC migration (Figure 6J, lanes 3 and 4 compared with lane 2), and subsequently to influence actin organization and distribution by increasing the ratio of F-actin to G-actin in VICs (Figures 6K,L, lanes 3 and 4 compared with lane 2).

**FIGURE 7:**
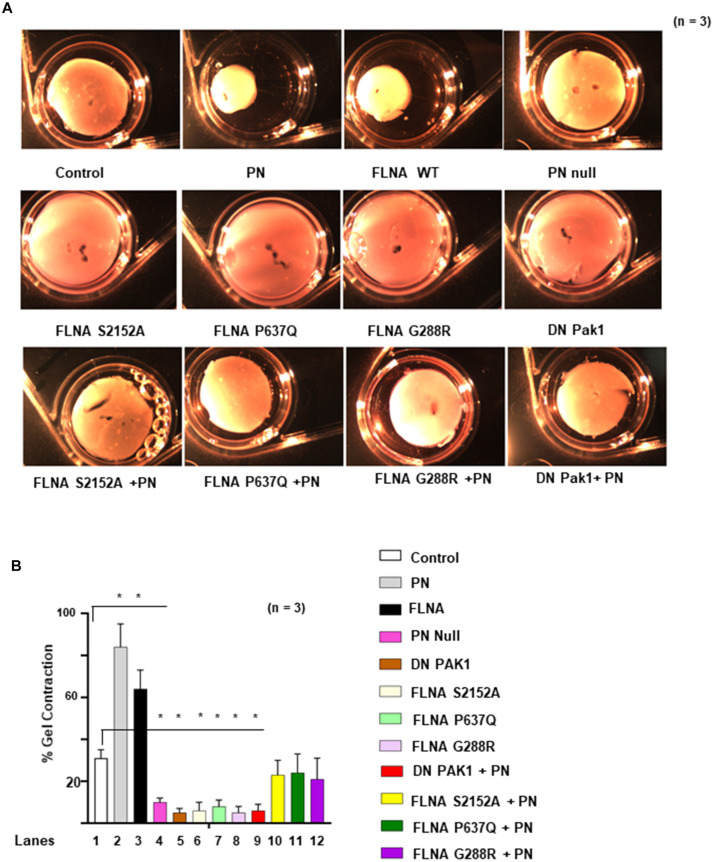
Effects of FLNA mutations [FLNA (P637Q) and FLNA (G288R)] on PN-induced cell differentiation measured by collagen gel compaction. **(A)** WT VICs were first transfected with the indicated constructs for 72 h before treatment with PN (1 μg/ml for 1 h). These transfected VICs and untransfected PN null VICs were then embedded in three-dimensional collagen matrices. **(B)** Matrix contractilities from the experiments in **(A)** were measured as % area of gel contracted and plotted in panel **(B)**. Results in panel **(A,B)** are representative of ≥3 separate experiments. Error bars represent SD. *p < 0.01 was considered significant.

Expression of a constitutively active Pak1 T423E mutant in FLNA-expressing wild-type VICs induced actin polymerization (Figure 2E, white bar in lane 4 compared with lane 1). PN stimulated this function of Pak1 T423E mutant in wild-type VICs (Figure 2E, black bar in lane 4 compared with lane 1). However, expression of this Pak1 T423E mutant in FLNA-deficient VIC Δ FLNA cells failed to induce actin polymerization in 80% of transfected cells (Figure 6M, white bar in lane 2 compared with white bar in lane 1). The stated defect in actin polymerization induced by the Pak1 T423E expression in the FLNA-deficient VIC Δ FLNA cells could be reversed by the cotransfection of FLNA-WT vector cells (Figure 6M, white bar in lane 3 compared with lane 1), and PN stimulated this actin polymerization (Figure 6M, black bar in lane 3 compared with white bar in lane 3). However, expression of FLNA mutants (FLNA S2152A, FLNA P637Q, and FLNA G288R) failed to save this impairment in actin polymerization as indicated in Figure 6M (white and black bars in lanes 4–6 compared with lane 3). These results suggest that actin polymerization mediated by PN requires interaction between active Pak1 and active FLNA. To assess whether these site-specific changes in the phosphorylation of FLNA and actin cytoskeleton assembly resulting from periostin/β-integrin signaling elicited functional changes that could promote collagen compaction, we employed collagen gel contraction assays to simulate what putatively occurs *in vivo* during valve remodeling (15). Figures 7A,B show that when VICs are established within a 3D collagen gel lattice and PN or FLNA signaling is initiated, the potential of these cells to contract (i.e., “compact”) the gel lattice is significantly increased compared with controls (Figures 7A,B, lane 2 and 3 compared with lane 1). This effect was abolished by transfecting the cells with mutant FLNAs (G288R, P637Q) (Figures 7A,B, lanes 7–8 compared with lane 3). Results using VICs isolated from WT VICs transfected with DN Pak1 or S2152A mutants also exhibited an impaired contraction/compaction phenotype when placed into 3D collagen gel cultures (Figures 7A,B, lanes 5 and 6 compared with lane 3 and lane 1). Cotransfection of DN Pak1 and FLNA (G288R, P637Q, and S2152A) mutants with wild-type PN cDNA minimally reversed (10–15%) the impairment of collagen compaction due to these inhibitory constructs (Figures 7A,B, lanes 9–12 compared with lane 1 and lane 3). These results indicate that the inductive effects of periostin on downstream signaling cascades that lead to the 2152 phosphorylation of FLNA have major effects upon valve compaction and maturation. They further indicate that an interaction of PN with its β-integrin receptors can induce activation of a Rac1-Cdc42-mediated, bidirectional Pak1-FLNA signaling pathway that promotes valve morphogenesis by regulating changes in actin cytoskeletal assembly, migration, and differentiation of valve interstitial progenitor cells.

## Discussion

Based on the periostin null mouse phenotype, studies show that PN impacts development of both inlet and outlet (arterial) valves and the primary atrial septum (Norris et al., 2008b; Snider et al., 2008; Markwald et al., 2010). However, in this study, we have focused primarily on the cellular progenitors of the inlet mitral valve, which, in most nulls, have an incomplete or absent anchoring fibrous annulus and abnormally shortened and thickened leaflets characterized by the lack of cell: matrix organization (Norris et al., 2008b; Snider et al., 2008). The interstitial cells of PN null valve leaflets are poorly differentiated or abnormally differentiated interstitial cells as indicated by the presence of cardiomyocytes, chondrocytes, or osteogenic cells (Norris et al., 2008b; Tkatchenko et al., 2009). This is in stark contrast to normal valves that are elongated and attenuated, and possesses an organized histoarchitecture consisting of thin layers of collagenous matrix and interstitial cells (Hajdu et al., 2011). Thus, we propose that the roles of PN in normal valve morphogenesis are to promote distal elongation and fibrogenesis by directing the differentiation of valve mesenchymal progenitor cells into fibroblasts that secrete and organize a collagenous matrix into compact, mature leaflets.

To assess the mechanisms by which PN regulates valve morphogenesis, we focused on signaling mechanisms that could be activated by PN interaction with its integrin receptors. Specifically, we found that PN, through its integrin receptors, can activate (phosphorylate) FLNA, a cytoskeletal protein that can bind to actin to generate morphogenetic contractile forces. That FLNA can generate sufficient forces to compact valve primordia into mature valves is supported by the mitral valve phenotype of patients with mutations in FLNA. Over time, the mitral leaflets of these patients degenerated and prolapsed due to loss of cell matrix organization (Duval et al., 2015; Levine et al., 2015; Sauls et al., 2015). How PN triggers activation of FLNA in valve progenitor cells and the functional significance of this activation on valve morphogenesis and maturation were the principal purposes of the present study (Ohta et al., 1999; Bourguignon, 2008; Sanz-Moreno et al., 2008). Drawing upon previous studies, we were able to establish that PN binding to α5β1 integrin activated a Fak/PI3K/Akt signaling pathway in valve mesenchyme progenitors cells (Ghatak et al., 2014), a finding that was recently confirmed in a study by Matsuzawa et al. in periodontal ligament fibroblasts (Matsuzawa et al., 2015).

In the present study, we further established that a downstream target of this PN/α5β1-integrin/Fak/Cdc42/Pak1 signaling is FLNA, which interacts directly with Pak1. We found that PN-induced bidirectional interaction between FLNA (S2152) and Pak1 in VICs is essential for actin rearrangement/migration/differentiation functions as occurs in postnatal valve maturation. Evidence for this conclusion is based on the following.

First, we found that PN, through specific integrin receptors, regulated the level of Cdc42-GTP and Rac1-GTP (Figures 2A,B). These members of the Rho subclass of the Ras superfamily of small molecular weight GTPases are known to transduce a variety of signals regulating many different cellular processes including cell cycle progression and cytoskeletal actin organization and contraction (Hall, 1998). For example, Rac1, when activated by agonists (e.g., platelet-derived growth factor or insulin) acts as a molecular switch that leads to the assembly of actin filaments at the cell periphery resulting in the formation of membrane protrusions such as lamellipodia or ruffles (Ridley and Hall, 1992), whereas activation of Cdc42 has been shown to induce actin-rich extensions of the surface protrusions called filopodia (Ridley and Hall, 1992; Kozma et al., 1995; Nobes and Hall, 1995). Thus, our finding that members of the GTPase family are responsive to signals initiated by PN binding provides a candidate mechanism by which this matricellular protein can trigger changes in the organization of the actin cytoskeleton of valve interstitial progenitor mesenchymal cells that are linked to valve differentiation and matrix compaction.

Second, we found that PN was activated by Cdc42 (GTP-bound state) through a FAK-Src/Pak1 kinase pathway that specifically induced Pak1 binding to FLNA (Figures 2–7). The strong interaction between Pak1 and FLNA proteins was observed after stimulation with physiological signaling molecule PN (Figures 5C, 6F). Thus, in VICs, the ability of PN to regulate the interaction of FLNA with Pak1, a downstream effector of the small GTPases, raises the possibility that FLNA, through its ability to bind to signaling molecules, may serve as a platform for small GTPase activities that connects PN/β-integrin in valve morphogenesis to trigger the cytoskeletal signals through activation of Pak1. Indeed exogenous treatment with cytochalasin D also inhibited the PN-induced interaction of Pak1 with FLNA, which indicates that an intact cytoskeletal structure is necessary for the PN-mediated interaction of Pak1 and FLNA. Furthermore, the PN-regulated Cdc42/Rac GTPases are required for interaction of FLNA with Pak1, which combined with the ability of FLNA to activate Pak1 kinase activity (Figure 6G) indicates that PN-associated FLNA may be involved in activating Pak1 in VIC migration/cytoskeleton structure and actin polymerization (Figures 6J–M). Importantly this interaction is necessary for differentiation of immature valve interstitial cushion cells into mature mitral valve leaflets (Figures 7A,B). Thus, it is not surprising that FLNA and PN expressions peak during late fetal and neonatal life when valve mesenchymal cells normally complete their fibrogenic differentiation into interstitial cells and are compacted into attenuated leaflets or cusps along with their secreted matrix (Butcher and Markwald, 2007; Kruithof et al., 2007; Norris et al., 2010).

Third, we found that the two point mutations in FLNA (G288R and P637Q) that are known to cause degenerative, myxomatous changes in patients that lead to mitral valve prolapse (Sauls et al., 2015) alter the potential of PN to induce the downstream phosphorylation of FLNA at S2152, which normally occurs through a Pak1-dependent events (Figures 6F,G). One significance of phosphorylating FLNA is that it promotes cytoskeletal actin arrangements that affect morphogenetic processes such as migration and differentiation. How it does is suggested in Figures 6H,I, which show that the G288R and P637Q point mutations in FLNA act to deregulate the balance between Rac1 and Rho GTPase activities, which correlates with reduced migration and FLNA–actin interaction in VICs (Figures 6J–M). These findings were consistent with the location of the G288R and P637Q mutations in the Igl repeats 1 and 4 of FLNA, respectively, which may contribute to a secondary interaction domain required for high avidity binding to F-actin (Nakamura et al., 2007; Sawyer and Sutherland-Smith, 2012). We found that the lower Rac1 activities (Figure 6H) observed in valve cells transfected with FLNA having P637Q and G288R mutations reduced F-actin/FLNA interaction (Figures 6K–M) and their potential to contract collagen gels compared with WT-FLNA-transfected valve cells (Figures 7A,B). The RhoA and Rac1 interaction domains on FLNA are in C-terminal repeats (Igl 21–23), which are remote from those targeted by the mutations. However, because FLNA is known to interact with RhoA and Rac1 (Ohta et al., 1999), it is possible that the mutations alter the spatial conformation of FLNA and its potential to bind to and organize the structure of the actin cytoskeleton.

## Conclusion

**FIGURE 8:**
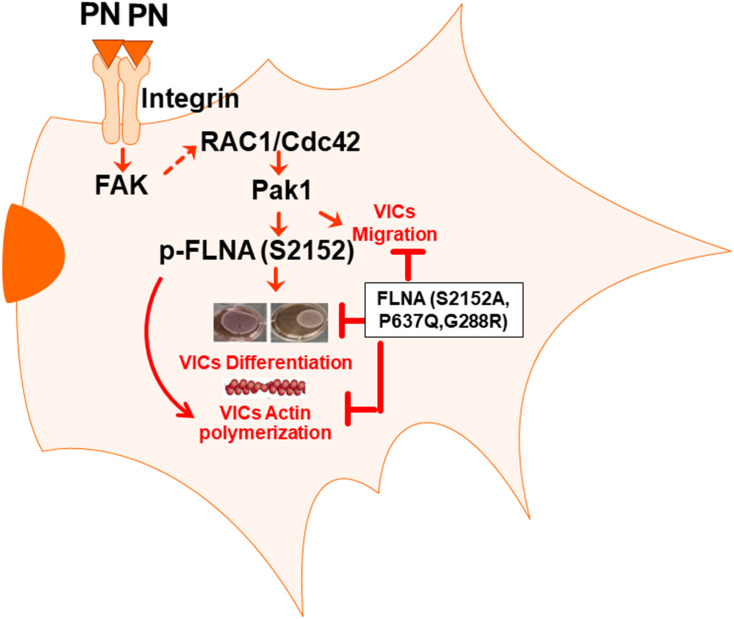
Model for the role of PN/α5β1 integrin-mediated Fak-Rac1-Cdc42-Pak1-FLNA signaling in VIC survival and remodeling functions. The figure summarizes our findings in a model that proposes how periostin and FLNA function as a central regulatory axis for valve morphogenesis. Overall, we conclude that a PN/α5β1 integrin interaction with FLNA engenders a unique signaling pathway that is transduced through the activation of Fak1/S6K signaling to Cdc42-Pak1-dependent activation of FLNA (Figures 1–7). This results in changes in adhesion and cytoskeletal organization that are linked to morphogenetic remodeling and maturation of valve primordia.

Collectively, our findings point to a central role for FLNA as an integrator that connects PN/β1 integrin signaling pathways to changes in cytoskeletal organization and function during pre- and postnatal valve morphogenesis. Our interpretation of the data presented in Figures 1–7 is summarized in a working model shown in Figure 8. In this model, a novel role for PN is indicated that triggers downstream activation of the effector protein, FLNA, which promotes changes in cytoskeletal assembly and organization required for valve development and postnatal maturation. Specifics for this model include: (1) PN/α5β1 integrin interaction triggers a FAK/Src signaling (Ghatak et al., 2019) pathway that promotes Cdc42/Pak1-mediated actin polymerization [shown in Green circle (Ghatak et al., 2019)] and activation of FLNA (S2152). (2) Additionally, the PN/β-Ig/FAK/Src signaling pathway also activates FLNA through a Rac1-dependent mechanism. (3) Both Pak1 and Rac1-mediated activation of FLNA promotes functional changes in the organization and function of the cytoskeleton that generate morphogenetic, contractile forces required to remodel valve primordia into compact, mature leaflets. (4) Two point mutations (P673Q and G228R), which are found in patients with degenerative (myxomatous) valve diseases, alter the balance of the small GTPases, RhoA and Rac1, and alter FLNA-Pak1 bidirectional interaction in response to PN to remodel the actin cytoskeleton assembly during cell adhesion, spreading, migration, and differentiation, thereby identifying candidate mechanisms that may contribute to the pathogenesis of mitral valve prolapse (Figures 1–7).

## Data Availability Statement

The datasets presented in this study can be found in online repositories. The names of the repository/repositories and accession number(s) can be found in the article/supplementary material.

## Ethics Statement

The animal study was reviewed and approved and done by all animal care and experiments in accordance with the institutional guidelines with IACUC-2017-00250 (approval date: 2019/03/14-2021/03/29).

## Author Contributions

SM and SG designed and carried out the experiments. SM, SG, and RM wrote the manuscript. VH reviewed and edited the multiple versions of the drafts and final versions of the text, figures, and figure legends. RM supplied all the reagents. RM-R and RN performed the immunohistochemistry of the heart sections. All authors contributed to the article and approved the submitted version.

## Conflict of Interest

The authors declare that the research was conducted in the absence of any commercial or financial relationships that could be construed as a potential conflict of interest.

## Footnotes

1 www.ncbi.nlm.nih.gov

## Abbreviations

VICs: valvular interstitial cushion fibroblast cells
PN: periostin
MBP: myelin basic protein
Fak: focal-adhesion-kinase
Pak1: p21-activated kinases
mTOR: mammalian target of rapamycin
p70S6K: p70 ribosomal protein S6 kinase
FLNA: filamin A.
